# PD‐L1‐Targeting Biomimetic Photoresponsive Thermosensitive Liposomes for Triple‐Negative Breast Cancer

**DOI:** 10.1002/advs.202506841

**Published:** 2025-08-11

**Authors:** Manman Tan, Chengyu Shi, Guangyi Chi, Xinwan Su, Fangzhou Liu, Linyu Zhu, Guangqian Cheng, Xiangyi Chen, Meng Yu, Yijian Chen, Ying Wang, Yu Chen, ShuLing Yan, Wenfei Wu, Qingfeng Yan, Jianzhong Shao, Kai Wang, Xiangrui Liu, Min Zhou, Aifu Lin

**Affiliations:** ^1^ MOE Laboratory of Biosystem Homeostasis and Protection College of Life Sciences Zhejiang University Hangzhou Zhejiang 310000 China; ^2^ Key Laboratory of RNA Science and Engineering Institute of Biophysics Chinese Academy of Sciences Beijing 100101 China; ^3^ Department of Respiratory and Critical Care Medicine Center for Oncology Medicine the Fourth Affiliated Hospital of School of Medicine and International School of Medicine International Institutes of Medicine Zhejiang University Zhejiang 322000 China; ^4^ Zhejiang Key Laboratory of Precision Diagnosis and Treatment for Lung Cancer Yiwu 322000 China; ^5^ Key Laboratory of Cancer Prevention and Intervention The Second Affiliated Hospital Zhejiang University School of Medicine Hangzhou Zhejiang 310000 China; ^6^ Department of Pharmacology, and Department of Gastroenterology of the Second Affiliated Hospital Zhejiang University School of Medicine Hangzhou Zhejiang 310000 China; ^7^ Future Health Laboratory Innovation Center of Yangtze River Delta Zhejiang University Jiashan Zhejiang 314102 China; ^8^ Zhejiang University‐University of Edinburgh Institute Zhejiang University School of Medicine Haining Zhejiang 314400 China; ^9^ Cancer Center Zhejiang University Hangzhou Zhejiang 310000 China; ^10^ Zhejiang Key Laboratory of Cell and Molecular Intelligent Design and Development Zhejiang 310000 China

**Keywords:** immunotherapy, light‐control, liposomes, peptide drug, triple‐negative breast cancer

## Abstract

Peptide‐based therapeutic strategies offer considerable potential for tumor immunotherapy but suffer from poor systemic bioavailability, rapid plasma clearance, and limited tumor‐targeting efficiency. To address these challenges, a biomimetic, photothermal‐responsive liposomal delivery system was developed that enables precise delivery of immunotherapeutic peptides while enhancing the synergistic effects of photothermal therapy. This system enhances peptide stability through fluorination, disrupts post‐translational modifications of PD‐L1, and promotes its degradation, thereby amplifying the anti‐tumor immune response. The carrier core consisted of thermosensitive 1,2‐dipalmitoyl‐sn‐glycero‐3‐phosphocholine (DPPC) liposomes loaded with the low‐toxicity photosensitizer indocyanine green (ICG), which facilitated controlled peptide release via the photothermal effect. Simultaneously, mild photothermal stimulation induced immunogenic cell death (ICD), further strengthening anti‐tumor immunity. To enhance tumor targeting, extend systemic circulation time, and improve drug accumulation at tumor sites, the carrier surface was coated with a platelet membrane, which increased biocompatibility and promoted immune evasion. Notably, in vivo studies demonstrated that the developed bioengineering platform significantly suppressed tumor growth in both solid and diffuse malignant tumors while inducing persistent immune memory, thereby facilitating long‐term anti‐tumor immune responses. Collectively, this approach establishes a novel framework for integrating peptide drug delivery with photothermal therapy, offering a promising strategy for advancing tumor immunotherapy.

## Graphical abstract

Tan et al. developed a platelet membrane‐coated, thermoresponsive liposome for targeted, light‐controlled delivery of fluorinated peptides that promotes programmed death‐ligand 1 degradation, induces tumor immunogenic cell death, suppresses solid tumors and metastases, and triggers durable immune memory, offering a promising strategy for peptide‐based cancer immunotherapy.

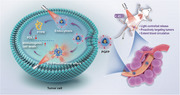

## 1 Introduction

Peptide‐based drugs are highly promising therapeutic modalities due to their superior specificity and selectivity in modulating physiological and pathological processes.^[^ ^1^ ^]^ By binding to specific cellular receptors or molecular targets, peptides have demonstrated significant potential in treating a wide range of diseases, including diabetes,^[^ ^2^ ^]^ infections,^[^ ^3^ ^]^ metabolic disorders,^[^ ^4^ ^]^ and cancer.^[^ ^5^ ^]^ In recent years, peptide‐based drugs have gained increasing attention in tumor immunotherapy, functioning by either blocking immune checkpoints or regulating post‐translational modifications.^[^ ^6^, ^7^ ^]^ For example, the peptide MOPD‐1 effectively inhibited tumor growth in CT26 tumor‐bearing mouse models by targeting PD‐L1 and disrupting the PD‐1/PD‐L1 interaction.^[^ ^7^ ^]^ Similarly, Han et al. developed a peptide that inhibited PD‐L1 palmitoylation, reduced its expression in cancer cells, and enhanced T‐cell‐mediated immunity against tumors.^[^ ^6^ ^]^ Transmembrane and ubiquitin‐like domain‐containing protein 1 (TMUB1), also referred to as hepatocyte odd protein shuttling (HOPS), is a highly conserved and widely expressed protein, initially identified during a screening for factors involved in rat liver regeneration.^[^ ^8^, ^9^ ^]^ TMUB1 participates in various biological processes, including inflammatory responses, genomic stability, apoptosis, cancer development, and cell proliferation.^[^ ^10^, ^11^, ^12^, ^13^ ^]^ In our previous study, we developed the competitive peptide—PTPR—consisting of 15 amino acids, which targets TMUB1, a key post‐translational modification regulator of PD‐L1. PTPR effectively downregulates PD‐L1 in tumor cells, enhances T cell‐mediated tumor cell destruction, and presents a promising strategy for augmenting anti‐tumor immunotherapy.^[^ ^14^ ^]^ Similar to many peptide‐based therapies, anti‐tumor immune peptides encounter significant challenges in clinical translation, such as low bioavailability, poor targeting specificity, and short in vivo half‐lives.^[^ ^15^ ^]^ In addition, due to their high polarity, hydrophilic peptide drugs face difficulties in penetrating cell membranes and reaching intracellular targets.^[^ ^16^ ^]^ These limitations reduce therapeutic efficacy and pose safety risks, such as immune‐related adverse events due to off‐target effects.^[^ ^17^ ^]^ Overcoming these delivery barriers and improving chemical modification and peptide drug delivery strategies are essential for advancing peptide‐based immunotherapies toward clinical application.

Several chemical modification strategies have been developed to improve the stability and delivery efficiency of peptide‐based drugs. In the late 1970s, Davis et al. first employed polyethylene glycol (PEG)‐modified bovine serum albumin (BSA), increasing its molecular weight and introducing steric hindrance to effectively protect amino acid residues.^[^ ^18^ ^]^ However, PEG modification can trigger immune responses, and the prolonged presence of PEG may lead to the development of “PEGylated immunity,” which accelerates blood clearance and gradually reduces drug effectiveness after repeated administration.^[^ ^19^, ^20^ ^]^ To address this issue, lipid modification strategies have been developed to reduce peptide polarity, enhance lipophilicity, and improve cell membrane permeability.^[^ ^21^, ^22^ ^]^ This modification significantly prolongs the half‐life of the drug, with lipid‐modified human growth hormone analogs exhibiting a half‐life of ≈40 h, enabling weekly administration.^[^ ^23^, ^24^ ^]^ Nonetheless, lipidation may increase the non‐specific distribution of the peptide, leading to its accumulation in non‐target cells and potentially causing systemic toxicity or side effects.^[^ ^25^ ^]^ Recent innovations include fluorination,^[^ ^26^ ^]^ phosphorylation,^[^ ^27^ ^]^ cyclic peptide N‐methylation,^[^ ^28^ ^]^ alpha‐helix structures,^[^ ^29^ ^]^ and other strategies aimed at enhancing peptide hydrolytic stability. Among them, fluorinated cationic polymers offer a promising solution for intracellular peptide drug delivery.^[^ ^30^ ^]^ Their stable C‐F bonds confer biological inertness, minimize non‐specific interactions with biomolecules in vivo, and reduce non‐specific adsorption.^[^ ^31^ ^]^ Their hydrophilic properties prevent excessive fusion with the cell membrane during internalization, reduce premature drug release, and minimize cytotoxicity.^[^ ^32^, ^33^ ^]^ Meanwhile, their lipophilicity enhances membrane permeability and facilitates endosomal escape, ensuring effective peptide delivery to the cytoplasm while preserving biological activity.^[^ ^31^, ^32^, ^34^ ^]^ The development of fluorinated labels to enhance the stability and efficiency of intracellular delivery of peptide drugs is a promising avenue for advancing peptide‐based immunotherapies.

In addition to chemical modifications, the development of nanocarrier systems offers a reliable solution for peptide drug delivery. Among these, liposomes offer clinical advantages due to their excellent peptide‐loading capacity, low immunogenicity, and minimal side effects.^[^ ^35^ ^]^ Nevertheless, traditional liposomes are often limited by their rapid clearance from the bloodstream and lack of specific targeting, resulting in inefficient drug release at the tumor site.^[^ ^36^, ^37^ ^]^ Furthermore, nanocarriers may undergo protein adsorption,^[^ ^38^ ^]^ or non‐specific uptake in physiological environments,^[^ ^39^ ^]^ leading to immune reactions, increased toxicity, and abnormal tissue distribution. Therefore, optimizing biomimetic and stimulus‐responsive modification techniques is critical for improving and controlling the targeting and release properties of liposomal drug systems.^[^ ^40^, ^41^ ^]^ Biomimetic approaches, such as coating nanoparticles with cell membranes, can mitigate these issues.^[^ ^42^ ^]^ Among them, platelet membrane camouflage is notable as it retains the surface protein profile of its source cells, thereby endowing nanoparticles with enhanced affinity for tumors, damaged vascular systems, and pathogens.^[^ ^43^ ^]^ Simultaneously, it enables immune evasion and extends in vivo circulation time, outperforming traditional coatings, such as PEG or liposomes.^[^ ^44^ ^]^ The effective use of biomimetic modification strategies can significantly enhance the efficiency of peptide drug delivery via nanoparticles and further optimize their application in anti‐tumor immunotherapy.

The development of stimulus‐responsive systems has introduced new approaches for the precise release of peptide drugs. A variety of responsive nanomaterials have been reported recently that react to stimuli, such as pH,^[^ ^45^ ^]^ glutathione levels,^[^ ^46^ ^]^ ultrasound,^[^ ^47^ ^]^ and magnetic fields,^[^ ^48^ ^]^ thereby enhancing drug targeting accuracy. However, the changes in the acidic pH of the tumor microenvironment are not always uniform,^[^ ^49^ ^]^ and the glutathione levels vary significantly across different tumor types,^[^ ^50^ ^]^ posing challenges for internal stimulus‐responsive systems. Additionally, although magnetic field therapy and ultrasound irradiation are widely used, achieving highly precise targeting remains difficult, potentially affecting surrounding healthy tissues and leading to side effects.^[^ ^51^ ^]^ Among these, near‐infrared (NIR) light‐responsive systems are notable for their non‐invasiveness, spatiotemporal control, and ability to minimize damage to the surrounding healthy tissues.^[^ ^52^ ^]^ Through controlled laser irradiation, these systems enable accurate and tunable drug release with minimal off‐target effects.^[^ ^53^ ^]^ Notably, moderate heating (≈42 ℃) induced by photothermal therapy (PTT) has been shown to promote immunogenic cell death (ICD), thereby stimulating anti‐tumor immunity within the tumor microenvironment.^[^ ^54^ ^]^ Thus light‐responsive systems not only facilitate precise drug delivery but also enable the integration of immunopeptide therapy with PTT. A rationally designed peptide delivery system that enhances stability, improves tumor targeting, and enables precise and responsive drug release would represent a significant advancement in peptide‐based immunotherapy.

**Scheme 1:**
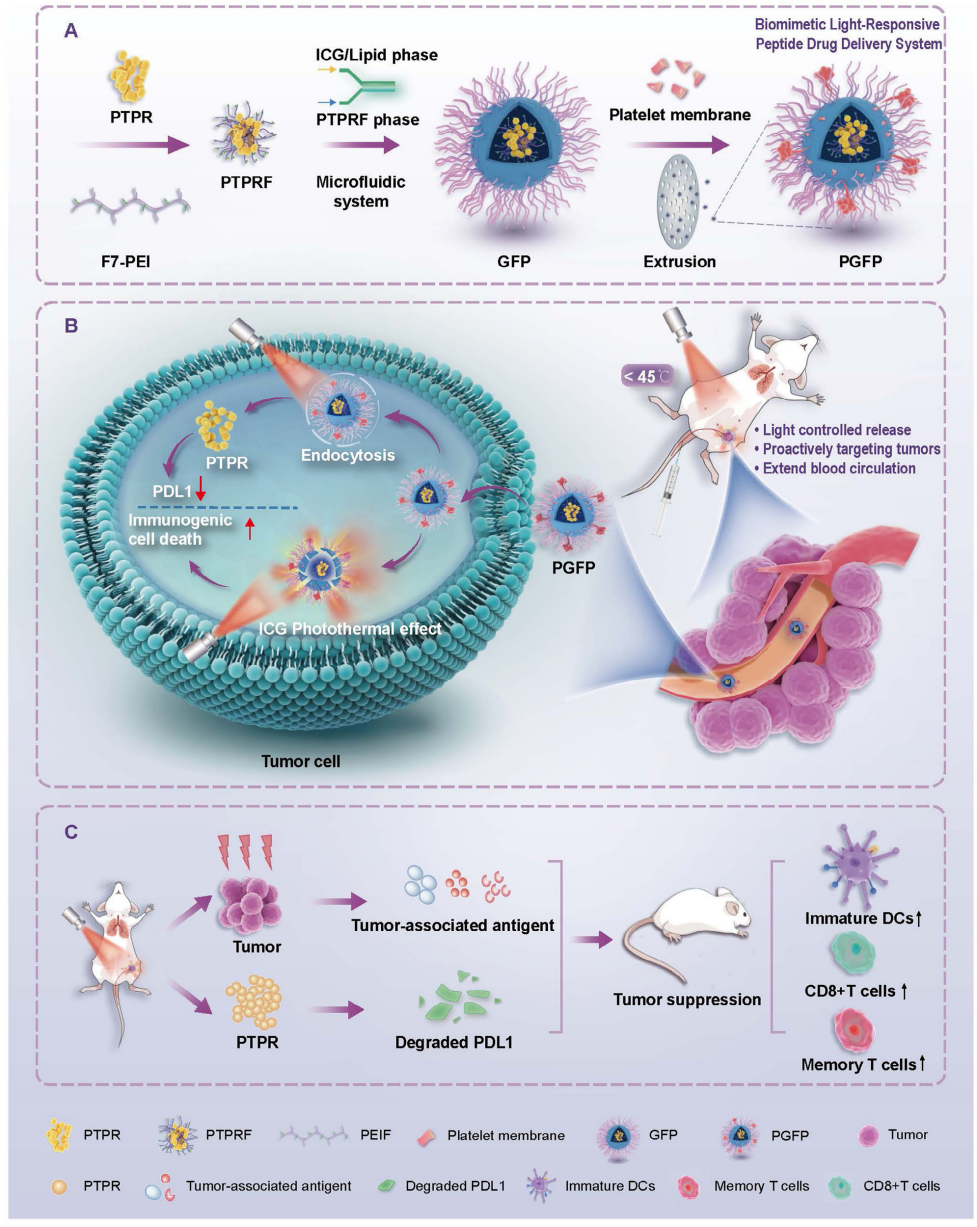
Schematic diagram of the photothermal immunotherapy strategy for cancer. A) Preparation method of PGFP nanocomplex. B) Schematic illustration of the synergistic mild photothermal ICD activation and PD‐L1 protein destruction treatment strategy. C) Photothermal activation of the PD‐L1 inhibition strategy reprograms the immunosuppressive tumor microenvironment.

In this study, we developed a photo‐controlled, temperature‐responsive hybrid biomimetic nanocarrier for the targeted delivery of the PD‐L1‐inhibiting peptide PTPR (**Scheme** **1**). This system features a thermosensitive liposomal core composed of 1,2‐dipalmitoyl‐sn‐glycero‐3‐phosphocholine (DPPC) and indocyanine green (ICG), enabling NIR‐triggered drug release. The fluorinated PTPR enhances PD‐L1 inhibition, while the outer biomimetic platelet membrane coating forms hybrid vesicles, prolonging systemic circulation and actively targeting tumors. Upon NIR irradiation, the system induces a tunable phase transition in the DPPC core, triggering peptide release and enabling mild photothermal therapy‐mediated ICD. In triple‐negative breast cancer models, including in situ, distal, and metastatic models, this system enhanced T‐cell infiltration, promoted dendritic cell maturation, stimulated systemic immune responses, and effectively downregulated PD‐L1 expression, thereby inhibiting tumor progression. Remarkably, it also induced durable immune memory responses in models of invasive tumors. Collectively, our study presents an integrated nanoplatform that combines precise tumor targeting and controlled drug release, offering a promising strategy for improving outcomes in peptide‐based cancer immunotherapy.

## 2 Results

### 2.1 Preparation and Characterization of In Situ Thermally Responsive PGFP

**Figure 1:**
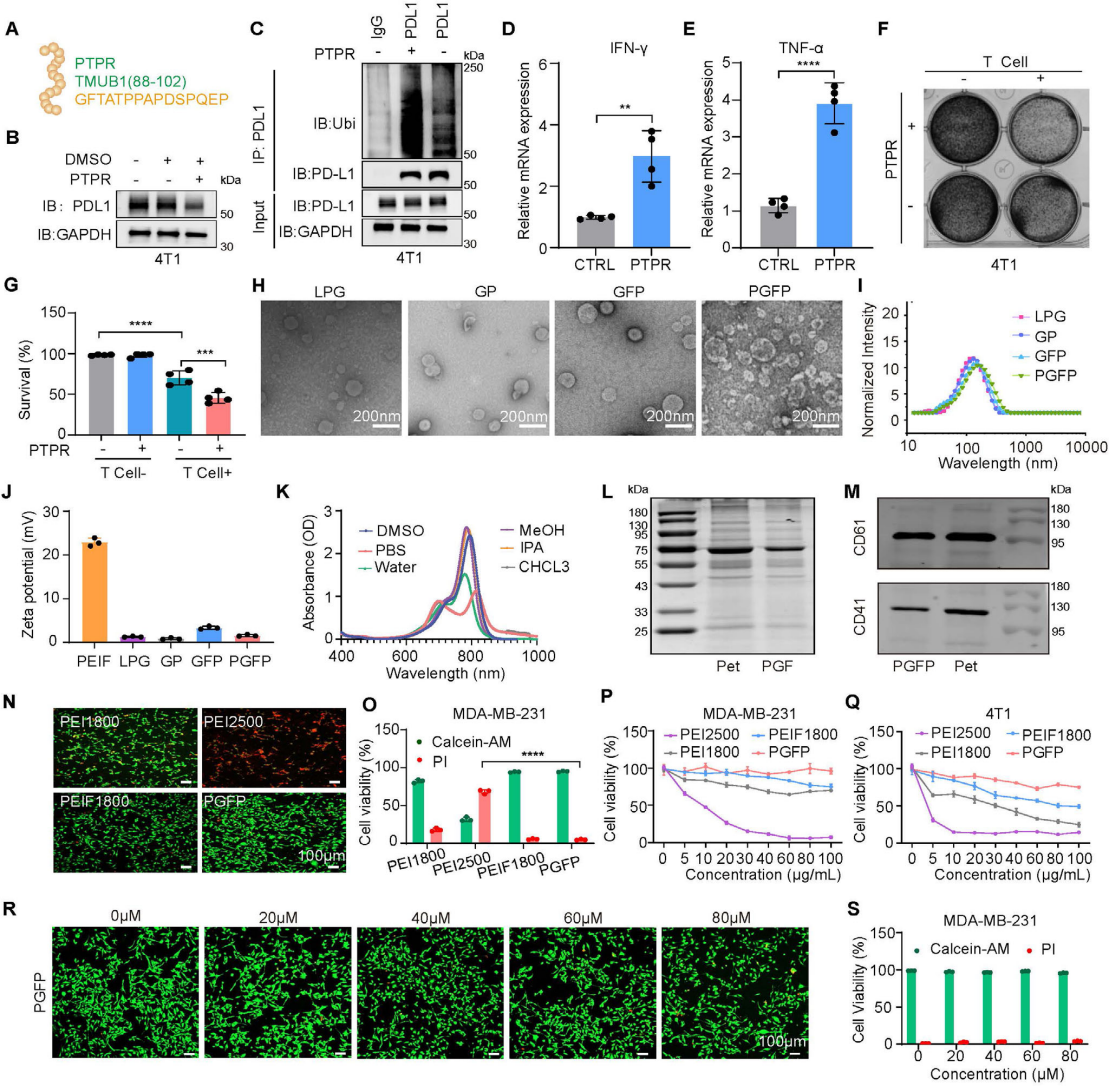
Preparation and Characterization of PTPR and PGFP. A) Illustration of the structure of the PTPR peptide. B) Immunoblot analysis of PD‐L1 expression in 4T1 cells. C) Co‐immunoprecipitation (Co‐IP) assay demonstrating the endogenous interaction involving PD‐L1. D,E) RT‐qPCR analysis of IFN‐γ (D) and TNF‐α (E) mRNA levels. The results are presented as mean ± SEM (n = 4). Two‐sided Student's t test. ****P < 0.0001, **P < 0.01. F,G) 4T1 cells were co‐cultured with activated T cells and then treated with PTPR or left untreated. Crystal violet staining was subsequently performed. The results are presented as mean ± SEM (n = 4). One‐way ANOVA with Tukey's test. ****P < 0.0001, ***P < 0.001. H) Typical TEM images of LPG (lipid nanoparticles loaded with ICG), GP (lipid nanoparticles co‐loaded with ICG and PTPR), GFP (lipid nanoparticles co‐loaded with ICG and fluorinated PTPR), and PGFP (lipid nanoparticles co‐loaded with ICG and fluorinated PTPR, camouflaged with platelet membranes), before and after hybridization with platelet membranes. I) Dynamic light scattering analysis of the size distribution of LPG, GP, GFP, and PGFP. J) Zeta potential characterization of LPG (liposomes loaded with ICG), GP, GFP, and PGFP. The results are presented as mean ± SEM (n = 3). K) Absorption spectra of PGFP in different media. L) SDS‐PAGE analysis of total membrane proteins isolated from purified platelet membranes and PGF. PGF indicates ICG‐loaded lipid nanoparticles hybridized with platelet membranes. M) Immunoblotting analysis of platelet‐associated proteins in purified platelet membranes and PGFP. N,O) Representative Calcein AM/PI staining images of MDA‐MB‐231 cells treated with 10 µg/mL PEI2500, PEI1800, PEIF1800, or PGFP, with corresponding ImageJ‐based quantification of cell viability and mortality. The results are presented as mean ± SEM (n = 3). Scale bar: 100 µm. One‐way ANOVA with Tukey's test. ****P < 0.0001 P,Q) CCK‐8 detection of cell viability in 231 and 4T1 cell lines treated with PEI2500, PEI1800, PEIF1800, and PGFP. The results are presented as mean ± SEM (n = 3). R,S) Calcein AM and PI staining images and fluorescence statistical analysis of MDA‐MB‐231 cells treated with different concentrations of PGFP. The results are presented as mean ± SEM (n = 3). Scale bar: 100 µm.

The peptide PTPR (TMUB1 PD‐L1 Regulatory Peptide; PTPR) (**Figure** **1**A) was developed in our previous study. PTPR disrupted the interaction between PD‐L1 and TMUB1 while simultaneously enhancing the binding of the E3 ubiquitin ligase HUWE1 to PD‐L1. This process facilitated PD‐L1 ubiquitination and degradation, leading to a reduction in the intracellular PD‐L1 levels in TMUB1‐overexpressing cells (Figure 1B,C). Furthermore, PTPR significantly enhanced T cell‐mediated cytotoxicity against tumor cells and upregulated the expression of TNF‐α and IFN‐γ in T cells (Figure 1D–G; Figure S1A,B, Supporting Information). These findings highlight the potential of PTPR in reducing intracellular PD‐L1 levels and restoring anti‐tumor immune responses.

To expand the therapeutic applications of PTPR, we developed a biomimetic, light‐responsive, and thermosensitive liposomal delivery system. Using microfluidics, the amphiphilic photosensitizer ICG and fluorinated PTPR (PTPRF) were co‐encapsulated into DPPC‐based thermosensitive liposomes. Specifically, the freeze‐dried fluorinated targeting peptide was uniformly distributed in the aqueous phase, whereas the amphiphilic ICG and DPPC lipids were dissolved in the ethanol phase, allowing precise control over drug encapsulation and release. Transmission electron microscopy (TEM) analysis revealed that, unlike non‐hybridized formulations, including LPG (liposomes loaded with ICG), GP (liposomes loaded with ICG and PTPR), and GFP (liposomes loaded with ICG and fluorinated PTPR), the platelet membrane‐hybridized PGFP (liposomes encapsulating ICG and fluorinated PTPR and coated with platelet membranes) exhibited a characteristic core–shell structure, indicating successful membrane fusion. Additionally, both nanoparticle formulations exhibited excellent dispersibility, with an average particle size of ≈100 nm (Figure 1H), suggesting their suitability for tumor accumulation and sustained drug retention.^[^ ^55^ ^]^ Dynamic light scattering (DLS) analysis confirmed that before platelet membrane hybridization, the nanoparticles had an average diameter of ≈105 nm, with a polydispersity index (PDI) of less than 0.25, indicating high uniformity and dispersion stability. After hybridization, the particle size of PGFP increased slightly to ≈120 nm, possibly due to the coating effect of the platelet membrane (Figure 1I; Figure S1C, Supporting Information). Zeta potential analysis revealed that PTPR is predominantly negatively charged under physiological conditions. At pH 6.5, the negative charge was significantly neutralized by the positive charge of PEIF after conjugation at a mass ratio of 10:1. Furthermore, LPG, GP, GFP, and PGFP exhibited weak charge characteristics compared to PEIF or PTPR alone, indicating that the liposomal or platelet‐derived outer membrane effectively shielded the surface charge of the nanoparticles (Figure 1J; Figure S2A–D, Supporting Information). The UV‐visible absorption spectra of PGFP in various media consistently displayed a peak at ≈785 nm, confirming that ICG retained its photothermal potential post‐encapsulation (Figure 1K; Figure S1D, Supporting Information). These findings show that the structural and functional integrity of the hybrid nanoparticles was effectively maintained, strongly supporting their potential for photothermal activation and biomedical applications.

To evaluate the protein composition of the platelet membrane in PGFP, SDS‐PAGE was performed on the platelet membranes and PGF (liposomes containing ICG and platelet membrane hybridization) before peptide drug encapsulation, followed by visualization using Coomassie brilliant blue staining (Figure 1L). The protein mass spectra of PGF closely resembled those of the platelet membrane, although the overall protein content was lower, possibly due to protein loss during extrusion. Immunoblotting confirmed the presence of specific protein markers, with key platelet surface proteins, such as CD41 and CD61, being detected in both PGFP and platelet membranes. These proteins are components of integrin αIIbβ3 and play essential roles in platelet adhesion and activation. Their presence confirms the successful transfer of platelet surface proteins to the functionalized PGFP nanoparticles, which is critical for achieving biomimicry and enhancing biocompatibility (Figure 1M). The peptide drug encapsulation efficiency of functionalized PGFP was assessed via fluorescence spectrophotometry. The results indicated that GP, GFP, and PGFP exhibited high peptide drug‐loading capacities (Figure S1E,F, Supporting Information). Cell viability was assessed using the CCK‐8 assay and Calcein AM/PI staining following PGFP treatment at various concentrations and different cell lines. The results demonstrated that LPG, GP, GFP, and PGFP exhibited negligible cytotoxicity in the absence of laser irradiation (Figure S1G,H, Supporting Information). Conversely, treatment with PEI2500 or PEI1800 significantly reduced the survival rates of MDA‐MB‐231 and 4T1 cells, underscoring the lower in vitro cytotoxicity of PGFP and fluorinated PEIF1800 (Figure 1N–Q; Figure S1I,J, Supporting Information). Furthermore, calcein‐AM/PI staining of MDA‐MB‐231 and 4T1 cells treated with various concentrations of PGFP demonstrated minimal in vitro cytotoxicity without NIR laser irradiation, supporting its safety and potential for therapeutic application (Figure 1R,S; Figure S1K,L, Supporting Information). Collectively, these findings demonstrate that PGFP possesses low inherent cytotoxicity, excellent drug‐loading efficiency, membrane‐derived targeting capabilities, and immunomodulatory features, providing a robust and multifunctional platform for targeted and stimuli‐responsive drug delivery.

### 2.2 In Vitro Cellular Uptake and Immunotherapeutic Efficacy of PGFP

**Figure 2:**
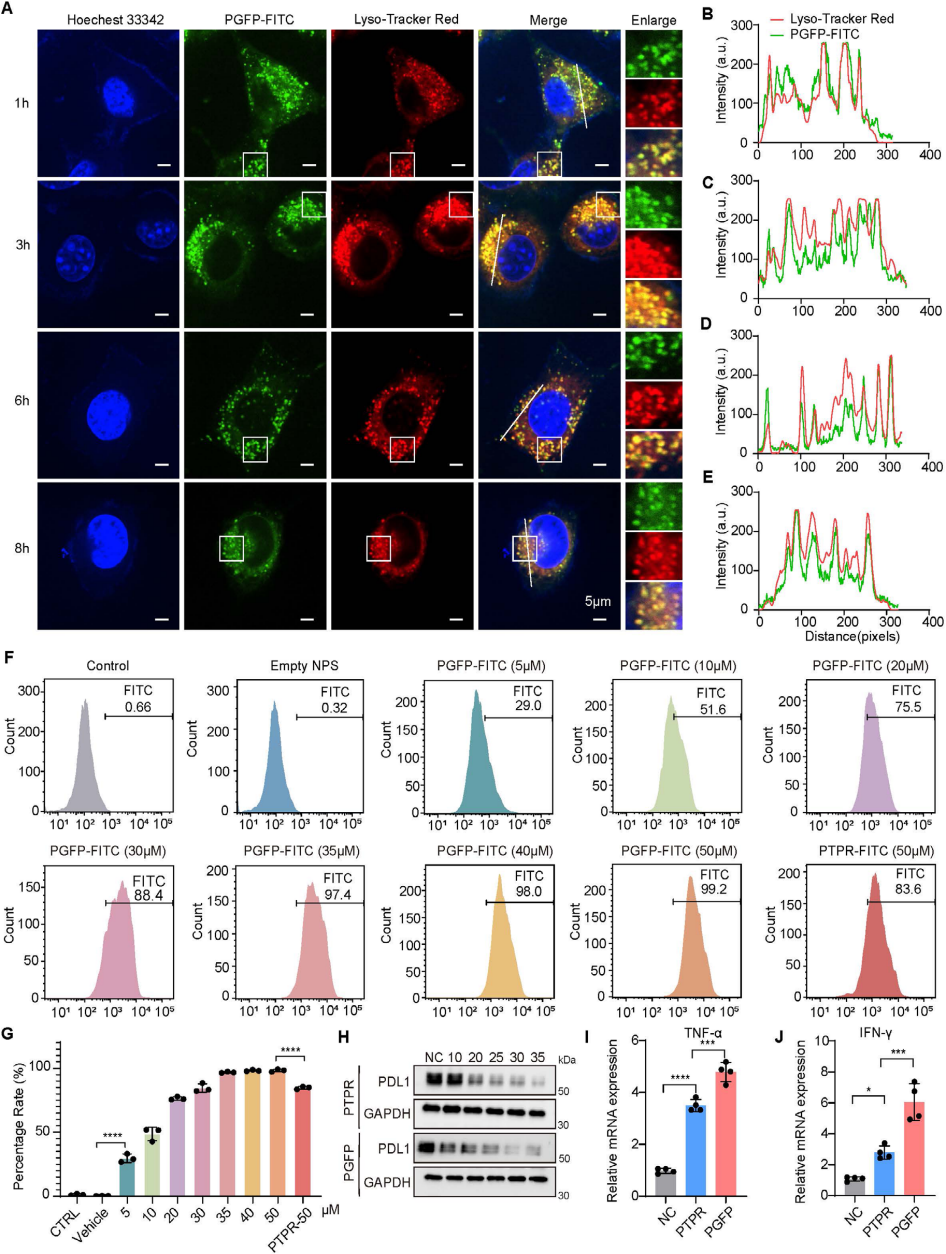
Cellular uptake and in vitro immune factors of PGFP. A–E) Confocal laser scanning microscopy images of 4T1 cells incubated with FITC‐labeled PGFP for 1, 3, 6, and 8 h. Endosomes were stained with LysoTracker Red, and nuclei were counterstained with Hoechst 33342. Scale bar: 5 µm. F,G) Flow cytometry analysis of cellular uptake, presented as the percentage of FITC‐positive cells. The results are presented as mean ± SEM (n = 3). One‐way ANOVA with Tukey's test. ****P < 0.0001 H) Immunoblot analysis showed the expression of PD‐L1 in 4T1 cells cultured for 48 h after exposure to PTPR or PGFP at 42 ℃ for 10 min. I,J) Expression of IFN‐γ (I) and TNF‐α (J) in T cells after PTPR or PGFP treatments was analyzed using RT‐qPCR. The results are presented as mean ± SEM (n = 4). One‐way ANOVA with Tukey's test. ****P < 0.0001, ***P < 0.001, *P < 0.05.

The FITC‐PGFP system was employed to assess the in vitro cytoplasmic delivery of PTPR. PGFP facilitates PTPR transport into the cytoplasm in a time‐dependent manner. Within the first hour of incubation, the majority of internalized PGFP co‐localized with LysoTracker Red, indicating endosomal localization (**Figure** **2**A,B). After 3–6 h of incubation without laser irradiation, a small fraction of PGFP had successfully escaped from the endosomes and was released into the cytoplasm (Figure 2C,D). By 8 hours, no additional endosomal escape was observed, indicating that cytoplasmic release of PGFP had reached completion (Figure 2E). Flow cytometry analysis of 4T1 cells treated with FITC‐PGFP revealed a dose‐dependent increase in PTPR (ranging from 10 to 50 µM), showing significantly higher uptake in PGFP‐treated cells compared to those treated with free FITC‐PTPR at 50 µM (Figure 2F,G). Such results confirm that PGFP substantially enhanced the delivery and cellular uptake of PTPR. Compared to GP and GFP, the PGFP nanoparticles coated with platelet membranes demonstrated markedly enhanced internalization by MDA‐MB‐231 cells (Figure S3A–H, Supporting Information). This enhanced uptake is likely attributable to the biomimetic properties of the platelet membrane. Specifically, platelet‐derived adhesion molecules such as P‐selectin, integrin α6β1, and integrin αIIbβ3 may engage tumor‐associated receptors such as PSGL‐1, CD44, ADAM9, and integrin αvβ3, ultimately promoting active internalization by tumor cells.^[^ ^56^ ^]^

Immunoblotting analysis indicated that PGFP and PTPRF effectively inhibited PD‐L1 expression at a concentration of 10 µm, whereas free FITC‐PTPR required a higher dose of 20 µM to achieve comparable inhibition (Figure 2H; Figure S2E, Supporting Information). This enhanced therapeutic effect may be attributed to the modification of fluorinated polyethyleneimine, which facilitates lysosomal escape. Furthermore, compared with LPG, GP, and GFP, PGFP not only enhanced T cell‐mediated cytotoxicity against tumor cells (Figure S3I,J, Supporting Information) but also showed a stronger PD‐L1 inhibitory effect (Figure S3K, Supporting Information), likely due to improved cellular uptake and endosomal escape facilitated by both the platelet membrane and fluorinated polymer. Moreover, PGFP significantly upregulated the expression of TNF‐α and IFN‐γ in T cells compared to treatment with PTPR alone (Figure 2I,J; Figure S3L,M, Supporting Information), highlighting its potential to enhance anti‐tumor immune responses. These findings demonstrate that PGFP not only improves the intracellular delivery of PTPR but also boosts its immunotherapeutic efficacy by effectively downregulating PD‐L1 and promoting T cell‐mediated cytotoxicity.

### 2.3 Photothermal Effect and Photothermal Activation Evaluation of PGFP

**Figure 3:**
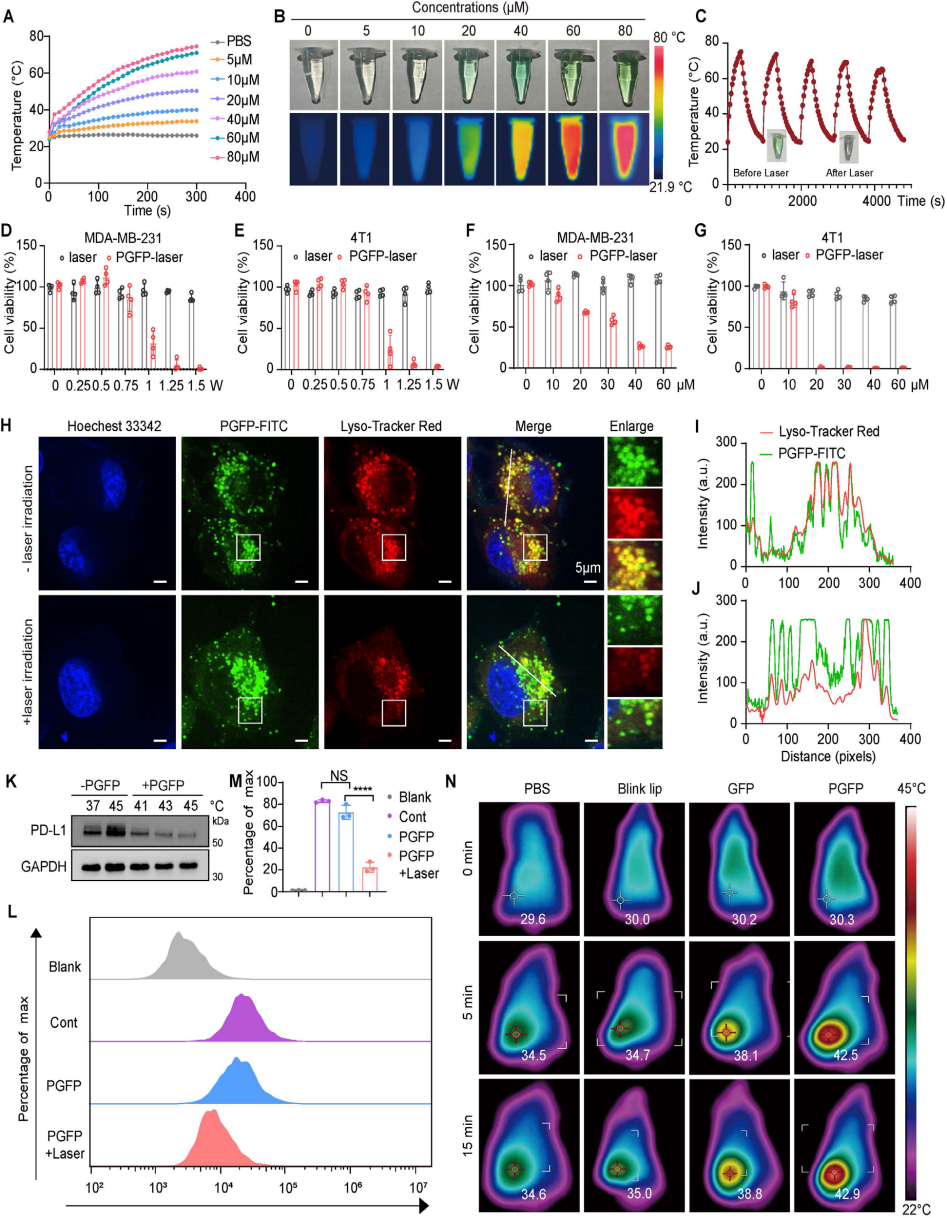
Evaluation of Photothermal Activation of PGFP In Vitro and In Vivo. A) Temperature rise curves of PGFP at various ICG concentrations under 1 W/cm^2^ of near‐infrared laser irradiation. B) Infrared thermal images of PGFP with varying ICG concentrations under 1 W/cm^2^ laser irradiation. C) Temperature fluctuation of PGFP over five consecutive on/off irradiation cycles at 1 W/cm^2^. D,E) Cell viability of MDA‐MB‐231 or 4T1 cells treated with PGFP under laser irradiation at various power densities (0‐1.5 W/cm^2^). The results are presented as mean ± SEM (n = 4). F,G) Cell viability of MDA‐MB‐231 and 4T1 cells treated with PGFP at varying ICG concentrations under 1 W/cm^2^ laser irradiation. The results are presented as mean ± SEM (n = 4). H–J) Confocal laser scanning microscopy (CLSM) images and localization analysis curves showing intracellular distribution of FITC‐PGFP complexes after 3 h of incubation, with and without near‐infrared irradiation.Scale bar: 5 µm. K) Immunoblotting analysis of PD‐L1 protein expression in response to PGFP treatment at different temperatures. L,M) Flow cytometry analysis of PD‐L1 protein inhibition by PGFP under conditions with and without near‐infrared illumination. The results are presented as mean ± SEM (n = 3). One‐way ANOVA with Tukey's test. ****P < 0.0001. N) Infrared thermal images of mice treated with PBS, blank liposomes, GFP, and PGFP under near‐infrared laser irradiation.

ICG is widely utilized in medical applications, such as blood circulation monitoring, liver function assessment, tumor detection, and lymph node imaging, owing to its excellent biocompatibility and strong NIR fluorescence properties.^[^ ^57^, ^58^ ^]^ To evaluate the photothermal effect and drug release behavior of the biomimetic PGFP nanocarriers, temperature changes were monitored under NIR irradiation at different PGFP concentrations. Under 808 nm laser irradiation (1.0 W/cm^2^), PGFP exhibited a concentration‐dependent increase in temperature, with a surge of over 30 ℃ occurring within 2 min at a concentration of 60 µM. Conversely, PBS showed minimal temperature changes under identical conditions (**Figure** **3**A,B). Additionally, PGFP (ICG 80 µM) exhibited robust photothermal stability across five consecutive on/off irradiation cycles, experiencing only a slight reduction in heating efficiency after repeated exposure while maintaining temperatures above 60 ℃ (Figure 3C). To evaluate its photothermal performance under mild irradiation conditions, the temperature variations of PGFP (60 µM) were measured at different power densities. At 0.33 W/cm^2^ for 5 min, the temperature rose to 42–45 ℃ (Figure S4A, Supporting Information). Notably, such a temperature range did not significantly affect MDA‐MB‐231 and 4T1 cells viability, indicating its suitability for in vitro photo‐triggered drug release (Figure S4B,C, Supporting Information). Subsequently, the cytotoxic effects of photoactivated PGFP were evaluated in 4T1 and MDA‐MB‐231 cells. PGFP‐induced cytotoxicity was dose‐ and laser‐dependent, with elevated concentrations, prolonged irradiation, and higher power densities resulting in greater reductions in cell viability (Figure 3D–G; Figure S4D,E, Supporting Information). The lysosomal escape capability of PGFP under NIR irradiation was evaluated using PGFP nanoparticles labeled with DID (a far‐infrared fluorescent dye) or FITC‐PTPR in conjunction with the lysosomal markers LysoTracker Green or Red. After 3 h of incubation, the laser‐irradiated PGFP nanoparticles demonstrated a significantly enhanced ability to escape lysosomes compared to the non‐irradiated controls (Figure 3H–J; Figure S4F–H, Supporting Information). The increase in temperature induced by the laser (≈42 ℃) promoted the release of PTPR from the PGFP nanoparticles, disrupting the post‐translational modifications of PD‐L1 and leading to a reduction in its expression (Figure 3K–M). Such results underscore the potential of PGFP to facilitate precise laser‐triggered drug release, thereby improving the therapeutic efficacy of the PD‐L1 immune checkpoint blockade.

The in vivo photothermal performance was evaluated by injecting PGFP into the tail vein of mice, followed by tumor irradiation with NIR light after an 8‐h circulation period. Tumors in PGFP‐treated mice exhibited a rapid temperature increase under an initial laser power of 2.0 W/cm^2^, with the tumor temperature successfully maintained at ≈42 ℃ by adjusting the laser power (Figure 3N). In contrast, tumors in PBS, blink liposomes, or GFP‐treated mice showed slower temperature increases and lower maximum temperatures. Although GFP‐treated tumors displayed a rapid increase in temperature, they did not reach the same tumor temperature as that of the PGFP group. The enhanced photothermal performance of PGFP is likely attributable to the biomimetic platelet membrane coating, which improves tumor accumulation and photothermal conversion efficiency. These findings demonstrate that PGFP is a highly effective and controllable photothermal agent with significant tumor‐targeting and therapeutic potential.

### 2.4 CD62p/CD44‐Mediated Tumor Targeting of PGFP Nanocarriers In Vivo

**Figure 4:**
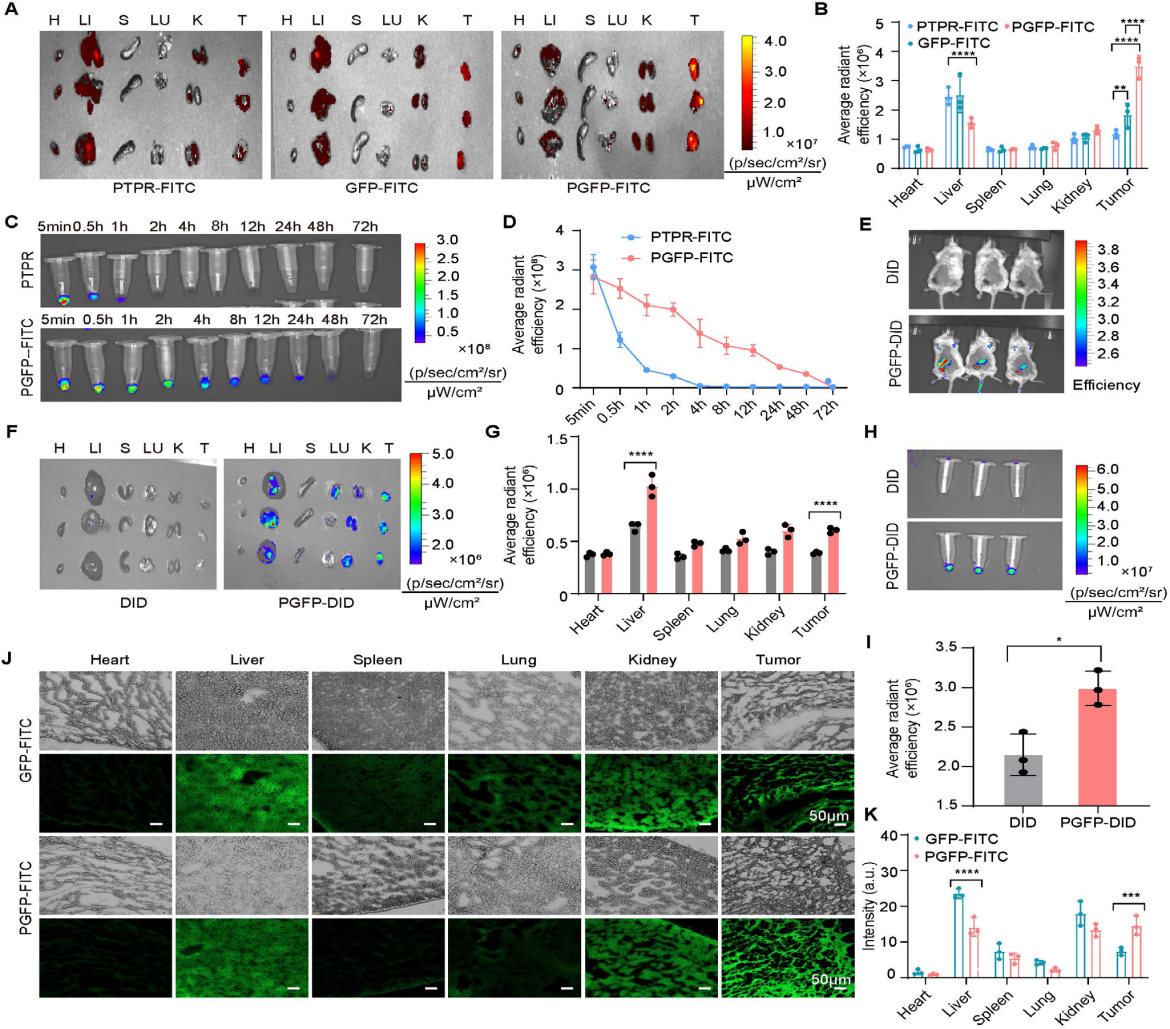
In Vivo Tumor Targeting Effect of PGFP. A,B) Ex vivo fluorescence imaging and quantitative analysis were conducted to assess the accumulation of FITC‐PTPR in tumors and major organs of 4T1 tumor‐bearing mice, 24 h after the intravenous injection of FITC‐PTPR, GFP‐FITC, or PGFP‐FITC. The results are presented as mean ± SEM (n = 3). Two‐way ANOVA with Tukey's test. ****P < 0.0001, **P < 0.01. C,D) The time‐dependent fluorescence intensity of FITC‐PTPR in mouse serum was measured following intravenous injection of either free FITC‐PTPR or PGFP‐FITC The results are presented as mean ± SEM (n = 3, blood samples per time point). E) In vivo fluorescence imaging of mice was performed 12 h after a tail vein injection of either free DID or DID‐labeled PGFP (n = 3). F,G) Ex vivo fluorescence imaging and quantitative analysis of DID accumulation were carried out in tumors and major organs of 4T1 tumor‐bearing mice. The results are presented as mean ± SEM (n = 3). Two‐way ANOVA with Tukey's test. ****P < 0.0001. H,I) Fluorescence imaging and quantitative analysis of DID fluorescence intensity in mouse serum were conducted 12 h after the intravenous injection of free DID or DID‐PGFP. The results are presented as mean ± SEM (n = 3, blood samples). Statistical significance was assessed using Student's t‐test. *P < 0.05. J,K) Representative fluorescence images and quantitative analysis of fluorescence intensity in frozen tumor sections and major organs were obtained 12 h after a tail vein injection of GFP‐FITC or PGFP‐FITC. The results are presented as mean ± SEM (n = 3). Scale bar: 50 µm. Two‐way ANOVA with Tukey's test. ****P < 0.0001, ***P < 0.001.

Fluorescently labeled nanocarriers (PTPR‐FITC, GFP‐FITC, or PGFP‐FITC) were intravenously injected into 4T1 tumor‐bearing BALB/c mice to evaluate the circulation dynamics and biodistribution. Imaging of tumors and major organs was conducted 24 h after tail vein injection, revealing that PGFP‐FITC and GFP‐FITC accumulated more in the tumor tissues than PTPR‐FITC, which exhibited relatively weak fluorescence. Notably, PGFP‐FITC modified with biomimetic platelet membranes demonstrated significantly stronger tumor accumulation than GFP‐FITC (**Figure** **4**A,B). This enhancement was attributed to the specific binding of the hybrid platelet membrane protein CD62p to the CD44 receptor, which is overexpressed on tumor cell surfaces.^[^ ^59^, ^60^ ^]^ These findings suggest that the CD62p/CD44 interactions effectively mediate the active targeting of PGFP to the tumor microenvironment. Fluorescence imaging of blood samples collected at various time points revealed that GFP‐FITC and PGFP‐FITC significantly prolonged the plasma circulation time compared to PTPR‐FITC (Figure 4C,D; Figure S5A,B, Supporting Information). Among the tested formulations, PGFP‐FITC exhibited the longest circulation time of ≈48 h (Figure 4D). These findings suggest that platelet membrane modification substantially extends nanocarrier circulation in the bloodstream, potentially enhancing their therapeutic efficacy.

In vivo imaging was performed to assess the DID‐PGFP biodistribution in mice. The DID‐PGFP signals were predominantly localized in subcutaneous tumor nodules near the liver and breast regions 12 h post‐injection (Figure 4E,F). Conversely, unencapsulated DID exhibited lower tumor localization and rapid systemic clearance (Figure 4G–I). Fluorescence analysis of frozen sections of the major organs (heart, lungs, kidneys, liver, and spleen) collected 12 h after FITC‐PGFP injection confirmed the reduced off‐target accumulation and increased tumor‐specific localization (Figure 4J,K; Figure S5C,D, Supporting Information). These findings indicate that the platelet membrane‐modified PGFP nanosystems have a significantly prolonged plasma half‐life and enhanced tumor accumulation, supporting their potential as an effective platform for targeted cancer therapy.

### 2.5 Anti‐Tumor Effect of PGFP Upon 808 nm Laser Irradiation

**Figure 5:**
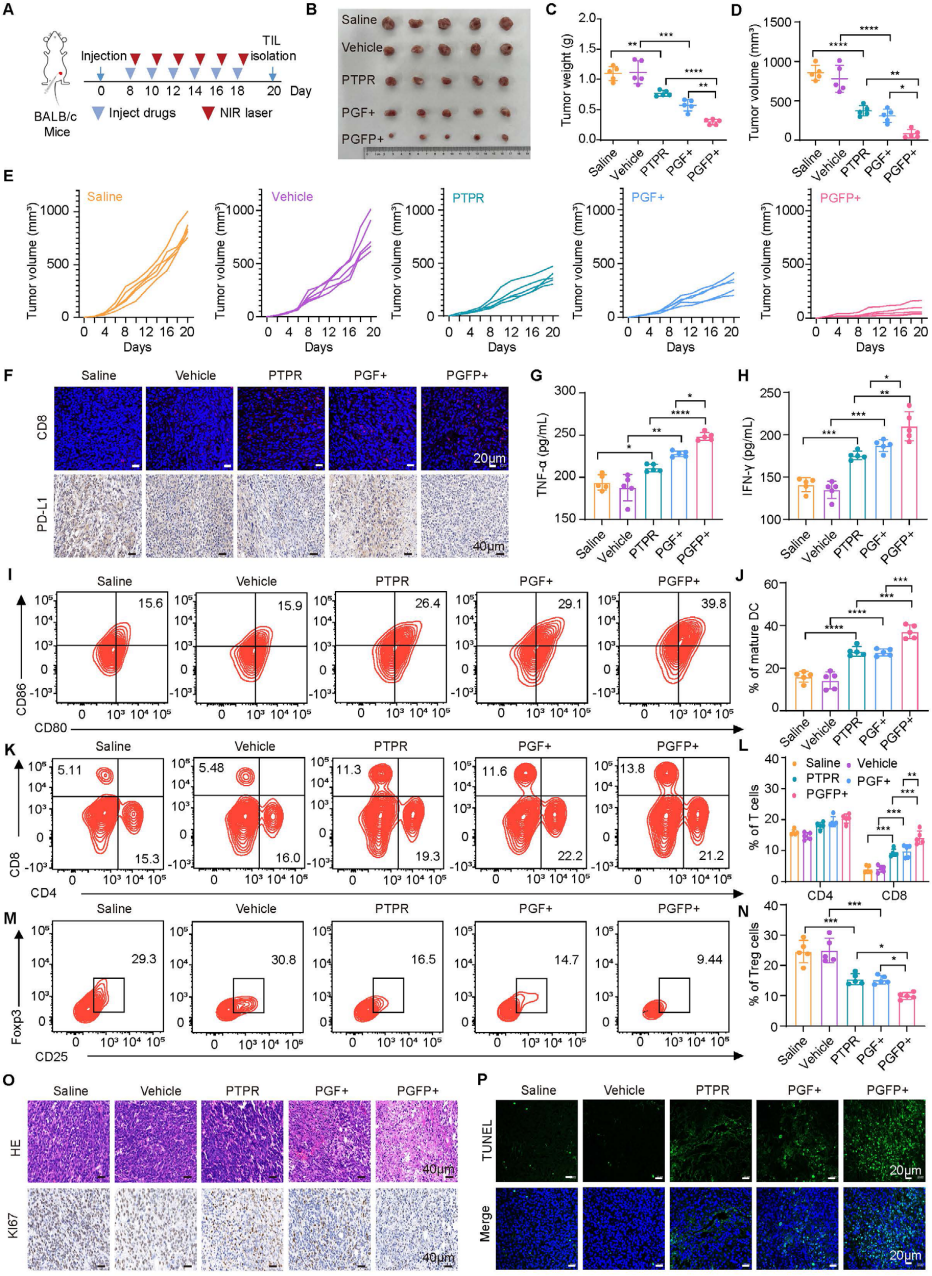
Mild Photothermal Therapy Combined with Immunotherapy Effectively Inhibits Primary Tumor Growth. A) A schematic diagram of the experimental design for the animal studies. B) Images of tumors from various treatment groups in 4T1 tumor‐bearing BALB/c mice (n = 5) following treatment. C) Statistics on tumor weight from mice subjected to different treatments. The results are presented as mean ± SEM (n = 5). One‐way ANOVA with Tukey's test. ****P < 0.0001, ***P < 0.001, **P < 0.01. D) Tumor volume statistics from mice post‐treatment. The results are presented as mean ± SEM (n = 5). One‐way ANOVA with Tukey's test. ****P < 0.0001, **P < 0.01, *P < 0.05. E) Growth curves of primary tumors for individual mice in the 4T1 tumor‐bearing BALB/c mouse model after various treatments. F) Immunofluorescence images of CD8^+^ T cells and immunohistochemistry images of PD‐L1 in tumor tissues from BALB/c mice after treatment. Scale bars: 20 or 40 µm. G,H) Plasma levels of TNF‐α and IFN‐γ in mice post‐treatment, measured by ELISA. The results are presented as mean ± SEM (n = 5). One‐way ANOVA with Tukey's test. ****P < 0.0001, ***P < 0.001, **P < 0.01, *P < 0.05. I,J) Analysis of dendritic cell (DC) maturation induced by the PGFP+ strategy in the 4T1 tumor‐bearing mouse model (gated on CD11c^+^ DC cells). Lymph node cells were collected after treatment and analyzed using flow cytometry with CD11c, CD80, and CD86 markers. The results are presented as mean ± SEM (n = 5). One‐way ANOVA with Tukey's test. ****P < 0.0001, ***P < 0.001. K,L) Flow cytometric analysis of CD4^+^ and CD8^+^ T cells from mouse spleens.The results are presented as mean ± SEM (n = 5). One‐way ANOVA with Tukey's test. ***P < 0.001, **P < 0.01. M,N) Flow cytometric analysis of Treg cell frequencies in the spleens of mice after treatment. The results are presented as mean ± SEM (n = 5). One‐way ANOVA with Tukey's test. ***P < 0.001, *P < 0.05. O,P) Representative confocal images of tumor tissue sections stained with H&E, Ki67, and TUNEL after treatment. Scale bars: 20 or 40 µm.

A 4T1 tumor‐bearing BALB/c mouse model was used to evaluate the anti‐tumor effects of PGFP combined with mild photothermal therapy, focusing on PD‐L1 disruption and ICD in solid tumors. The NIR laser treatment group received a single laser irradiation (808 nm, 42 ℃, 15 min) 8 h post‐injection (**Figure** **5**A). Compared with the saline, PTPR, and PGF+ (lipid nanoparticles coated with platelet membranes and co‐loaded with ICG and PEIF; “+” indicates near‐infrared laser irradiation) treatments, the combination of PTPR with nanocarrier‐mediated photothermal therapy (PGFP+) exhibited superior anti‐tumor effects (Figure 5B–E). Furthermore, we analyzed the immune cell activity in the spleen, lymph nodes, and tumors, along with the serum levels of key immune cytokines. The PTPR group showed increased infiltration of CD8⁺ T cells and reduced PD‐L1 protein expression in tumor tissues compared to the saline group. Similarly, mild photothermal therapy resulted in the upregulation of PD‐L1 protein expression and an increase in CD8⁺ T cell infiltration, suggesting an enhancement of ICD and a potential adaptive immune response. Notably, the PGFP+ group exhibited lower PD‐L1 protein expression and higher CD8⁺ T cell infiltration in tumor tissues than the PGF+ group (Figure 5F; Figure S6A,B, Supporting Information). Additionally, the serum levels of TNF‐α and IFN‐γ were significantly elevated in PGFP+‐treated mice compared to the PGF+ and PTPR groups (Figure 5G,H, Supporting Information), indicating robust immune activation. Furthermore, the proportions of mature dendritic cells (Figure 5I,J) and CD8⁺ cytotoxic T lymphocytes (Figure 5K,L; Figure S7, Supporting Information) were significantly higher in the PGFP+ group, whereas the proportion of regulatory T cells (Tregs) was notably lower (Figure 5M,N; Figure S7, Supporting Information). Corresponding to these alterations in tumor‐infiltrating lymphocytes, the PGFP+ treatment significantly inhibited tumor cell proliferation, promoted necrosis and apoptosis within tumor tissues, and markedly prolonged survival (Figure 5O,P; Figure S6C–E, Supporting Information). These results demonstrated that our delivery system successfully combined mild hyperthermia with PD‐L1 inhibition, substantially reducing tumor‐associated immunosuppression and enhancing anti‐tumor immune responses.

The in vivo safety of PGFP was also assessed, revealing no significant weight loss in any of the tested groups, indicating negligible toxicity (Figure S8A,B, Supporting Information). Histological analysis using H&E staining revealed no significant tissue damage or inflammatory lesions in the major organs (heart, liver, spleen, lungs, and kidneys) of PGFP+ ‐treated mice. These findings underscore the excellent biocompatibility and safety of PGFP and further validate its potential for photothermal immunotherapy.

### 2.6 PGFP Strategy Inhibits Distal Tumor Growth

**Figure 6:**
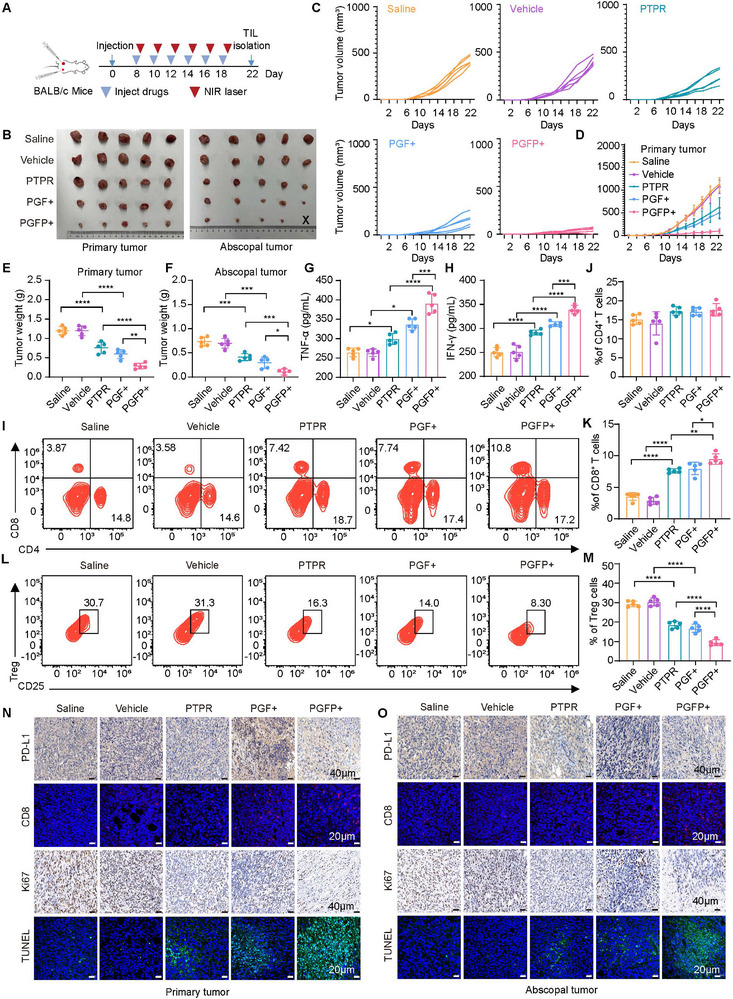
Therapeutic Effects of the PGFP+ Strategy on Distant Tumors. A) Schematic diagram of experimental design for distant tumors in mice. B) Ex vivo images of primary and distant tumor tissues from the 4T1 tumor‐bearing BALB/c mouse model (n = 5). C,D) Tumor growth curves for primary and distant tumors in the 4T1 tumor‐bearing BALB/c mouse model (n = 5). E,F) Tumor weights for primary and distant tumors in mice subjected to various treatments. The results are presented as mean ± SEM (n = 5). One‐way ANOVA with Tukey's test. ****P < 0.0001, ***P < 0.001, **P < 0.01, *P < 0.05. G,H) Serum levels of TNF‐α and IFN‐γ in mice after different treatments. The results are presented as mean ± SEM (n = 5). One‐way ANOVA with Tukey's test. ****P < 0.0001, ***P < 0.001, *P < 0.05. I–K) Flow cytometric analysis was conducted on CD4^+^ and CD8^+^ T cells in the spleens of mice following different treatments. The results are presented as mean ± SEM (n = 5). One‐way ANOVA with Tukey's test. ****P < 0.0001, **P < 0.01, *P < 0.05. L,M) Frequency of regulatory T cells (Tregs) in spleen tissues of mice after various treatments. The results are presented as mean ± SEM (n = 5). One‐way ANOVA with Tukey's test. ****P < 0.0001. N,O) Representative images of CD8^+^ effector T cells and TUNEL staining in tissue sections of primary and distant tumors. Immunohistochemical images of PDL1 and Ki67 protein expression in tumor tissue sections. Scale bar: 20 or 40 µm.

To investigate whether the PGFP‐induced active immune response could suppress distant untreated tumors, a dual‐tumor model was established by orthotopically injecting 4T1 tumor cells into the left and right mammary fat pads of mice. Only the primary tumor was irradiated, whereas the distant tumors remained untreated (**Figure** **6**A). Following treatment, the sizes of both the primary and distant tumors were monitored (Figure 6B–F; Figure S9A,B, Supporting Information). Notably, PGF+ treatment resulted in a reduced growth rate of distant tumors, underscoring its role in systemic immune activation. Importantly, the PGFP+ group exhibited significantly greater suppression of distant tumor growth compared to PGF+, emphasizing the synergistic effects of mild photothermal therapy and PD‐L1 inhibition in reshaping the “cold” tumor microenvironment.

Effector cytokine levels were measured in mouse serum to evaluate systemic immune activation. The levels of TNF‐α and IFN‐γ were significantly elevated in the PGFP+ group compared to the PTPR and PGF+ groups (Figure 6G,H). Moreover, combination treatment increased the proportion of CD8⁺ cytotoxic T lymphocytes and decreased the proportion of Tregs. Dendritic cell maturation was also significantly enhanced in the PGFP+ group (Figure 6I–M; Figure S9L,M, Supporting Information). These findings demonstrated that the PGFP+ strategy effectively induces systemic anti‐tumor immunity and promotes ICD. Additionally, the PGFP+ treatment led to increased infiltration and activation of cytotoxic T lymphocytes within distant non‐irradiated tumors (Figure 6N,O; Figure S9C,D, Supporting Information). Immunohistochemical analysis revealed significantly reduced PD‐L1 protein expression in distant tumors treated with the PGFP+ strategy. A similar decrease was also observed in the PGF+ group, possibly due to enhanced CTL‐mediated tumor killing (Figure 6N,O; Figure S9E,F, Supporting Information). Furthermore, distant tumors treated with the PGFP+ strategy showed an increase in TUNEL‐positive and necrotic cells, whereas the number of Ki67‐positive proliferating cells was significantly reduced (Figure 6N,O; Figure S9G–K, Supporting Information). These results suggest that the immune response in the distant tumor microenvironment was significantly enhanced, resulting in the effective suppression of distant tumor growth. Importantly, mouse body weights remained stable across all treatment groups, with no significant weight loss observed, further verifying the biocompatibility and safety of the PGFP strategy (Figure S9N, Supporting Information).

### 2.7 Anti‐Metastatic Tumor Activity of the Photoactivated PGFP System

**Figure 7:**
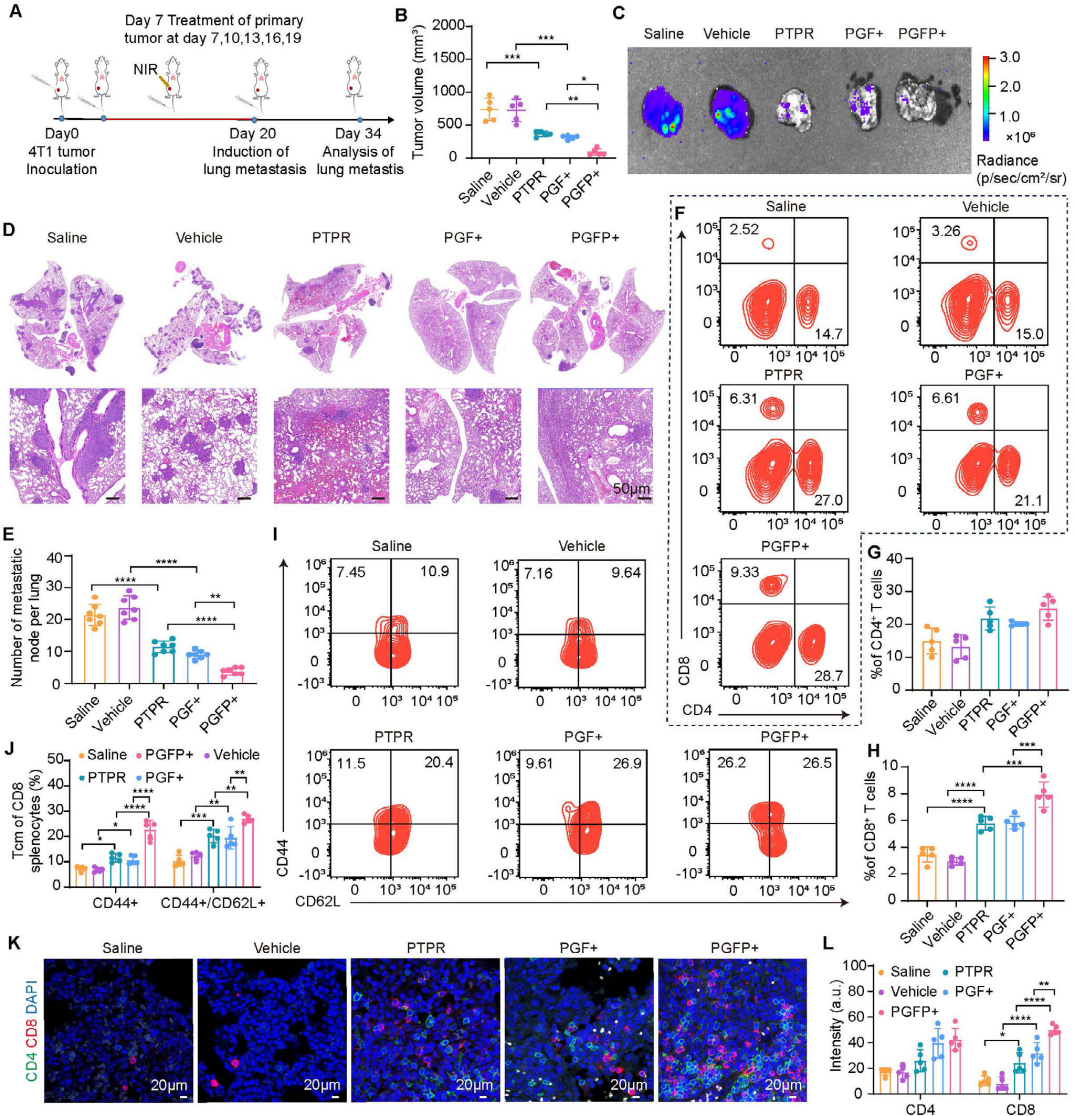
Prevention of Tumor Metastasis by PGFP+‐Induced Long‐Term Immune Effects in Mice. A) Schematic diagram illustrating the experimental design for PGFP+‐mediated inhibition of tumor metastasis. B) Statistics of the volume of isolated primary tumors in mice after treatments (saline, vehicle, PTPR, PGF+, PGFP+). The results are presented as mean ± SEM (n = 5). One‐way ANOVA with Tukey's test. ***P < 0.001, **P < 0.01, *P < 0.05. C) Bioluminescence imaging of lung metastatic nodules from 4T1 tumors after the indicated treatments. D) H&E staining of lung metastatic nodules from 4T1 tumors after the indicated treatments. E) Quantitative analysis of lung metastatic nodules. The results are presented as mean ± SEM (n = 7). One‐way ANOVA with Tukey's test. ****P < 0.0001, **P < 0.01. F–H) Flow cytometry analysis of CD4^+^ and CD8^+^ T cells in the spleen of mice following treatment. The results are presented as mean ± SEM (n = 5). One‐way ANOVA with Tukey's test. ****P < 0.0001, ***P < 0.001. I,J) Representative flow cytometry images and quantitative analysis of effector memory T cells (CD3^+^ CD8^+^ CD44^+^ CD62L^−^) and central memory T cells (CD3⁺CD8⁺CD44⁺CD62L⁺) in mice after the indicated treatments. The results are presented as mean ± SEM (n = 5). One‐way ANOVA with Tukey's test. ****P < 0.0001, ***P < 0.001, **P < 0.01, *P < 0.05. K,L) Representative immunofluorescence images and quantitative analysis of CD4^+^ (green) and CD8^+^ (red) T cells in 4T1 lung metastatic tumor tissue sections. The results are presented as mean ± SEM (n = 5). Scale bar: 20 µm. One‐way ANOVA with Tukey's test. ****P < 0.0001, **P < 0.01, *P < 0.05.

The long‐term immune memory effects induced by the PGFP+ treatment were assessed using a lung metastatic 4T1 tumor model with restimulation. In this model, luciferase‐labeled 4T1 cells were administered via tail vein injection into mice that had undergone specific treatments for two weeks (**Figure** **7**A). Only the primary tumors received irradiation, and changes in orthotopic tumor weights, ex vivo tumor volumes, and primary tumor weights were closely monitored. The combination of mild photothermal therapy and anti‐PD‐L1 peptide delivery effectively suppressed primary tumor growth and stimulated systemic anti‐tumor immunity (Figure 7B; Figure S10A–C, Supporting Information).

Bioluminescence imaging and H&E staining were performed to evaluate lung tumor metastasis and treatment efficacy in preventing recurrence. Mice pretreated with PGFP+ exhibited no discernible lung metastases, whereas those treated with saline or vehicle developed significant lung metastases within 14 d (Figure 7C–E). Flow cytometry analysis demonstrated an increased proportion of CD8⁺ cytotoxic T lymphocytes in the spleens of mice treated with PGFP+, PGF+, or PTPR, indicating a heightened systemic immune response (Figure 7F–H). Furthermore, the proportion of mature dendritic cells in the mesenteric lymph nodes was significantly higher in the PGFP+ group, further indicative of systemic immune activation (Figure S10D,E, Supporting Information). Memory T cell populations, including central memory T cells and effector memory T cells, were markedly elevated in the PGFP+, PGF+, and PTPR treatment groups (Figure 7I,J; Figure S11, Supporting Information), suggesting that the PGFP+ strategy promotes immune memory formation—essential for sustaining the anti‐tumor effects and preventing metastasis. Immunofluorescence analysis further revealed that the number of CD8⁺ T cells infiltrating metastatic lung tumors was significantly greater in the PGFP+, PGF+, and PTPR groups than in the saline or vehicle groups (Figure 7K,L). This finding indicates that mild photothermal therapy and PTPR treatment via the developed delivery system effectively enhanced cytotoxic T cell infiltration into metastatic tumors. ELISA analysis showed elevated serum levels of TNF‐α and IFN‐γ in the PGFP+ group, confirming robust immune activation (Figure S10F,G, Supporting Information). Collectively, these findings demonstrate that the PGFP+ approach activates systemic anti‐tumor immune responses, inhibits tumor cell metastasis, promotes memory T cell formation, enhances cytotoxic T cell infiltration, and facilitates dendritic cell maturation. Additionally, no significant weight loss was observed in any treatment group, demonstrating the excellent biocompatibility and safety of PGFP (Figure S10H, Supporting Information) and underscoring its significant potential for in vivo photothermal immunotherapy applications.

## 3 Discussion

The susceptibility of anti‐tumor immunotherapy peptides to plasma protein degradation, their short half‐life, poor intracellular delivery across cell membranes, and the immunosuppressive tumor microenvironment remain major obstacles to their effective systemic administration and therapeutic efficacy.^[^ ^15^, ^61^ ^]^ In this study, we integrated multiple regulatory strategies, including PD‐L1 inhibition, biomimetic‐targeted delivery, and photothermal‐responsive release, to offer novel approaches and overcome these challenges. Compared with existing PD‐L1 antibodies or small‐molecule inhibitors, PTPR peptides regulate PD‐L1 protein levels by targeting TMUB1‐PD‐L1 interactions, potentially circumventing tolerance issues caused by PD‐L1 translocation in conventional ICB therapy.^[^ ^14^ ^]^ The fluorination modification strategy used in this study improved the lysosomal release of the peptides and preserved their biological activity within the complex tumor microenvironment. Furthermore, the integration of platelet membrane coating technology significantly prolonged the circulation half‐life, with the CD62p protein on the platelet membrane specifically binding to CD44 receptors overexpressed on the tumor surface and mediating active tumor targeting.^[^ ^62^ ^]^ The mild photothermal effect mediated by ICG triggers DPPC liposomal phase transition, ensuring precise peptide drug release at the tumor site while preventing off‐target leakage. The controlled photothermal effect also promotes tumor antigen release, facilitates dendritic cell maturation, and enhances cytotoxic T cell activation and tumor infiltration. This synergistic immune‐enhancing mechanism significantly strengthened anti‐tumor immunity in both primary and metastatic triple‐negative breast cancer models. This strategy provides valuable insights into optimizing and advancing the clinical translation of peptide‐based immunotherapies.

Current anti‐tumor immunotherapy peptide combination therapies often face challenges related to limited spatiotemporal synergy. Although previous studies have demonstrated the effectiveness of photothermal therapy (PTT) combined with anti‐PD‐1/PD‐L1 therapy, most regimens rely on high temperatures (> 50 ℃) to induce ICD.^[^ ^63^, ^64^ ^]^ However, excessive heat can trigger non‐specific inflammatory responses and damage healthy tissues.^[^ ^65^ ^]^ Furthermore, when anti‐tumor immune peptides and photothermal agents are delivered via different carriers, their synergistic effects on heterogeneous tumor cell populations may diminish due to asynchronous drug distribution. Compounding this issue, PTT‐induced local hyperthermia can upregulate the expression of PD‐L1,^[^ ^54^ ^]^ while conventional PDL‐1 inhibitory peptides often fail to reach tumor lesions accurately and synchronously. In this study, we demonstrated that mild photothermal conversion (42 ℃) can effectively induce ICD while minimizing the risk of high‐temperature‐induced damage to healthy tissues. The developed biomimetic membrane‐targeted co‐delivery system incorporates a thermally controlled release mechanism, ensuring the synchronized action of the PTPR peptide and the PTT effect within the same tumor cell population. PTPR peptide‐mediated PD‐L1 degradation effectively counteracts PTT‐induced immune evasion, while the resulting ICD effect further amplifies T cell‐mediated tumor killing, establishing a positive immune feedback loop. This spatiotemporal collaborative optimization strategy has significant potential for inhibiting tumor growth and inducing long‐lasting anti‐tumor immunity. However, due to potential variations in the local photothermal conversion efficiency caused by tumor heterogeneity, integrating imaging‐guided temperature monitoring would be beneficial. For clinical translation, further investigation into pharmacokinetics and biological distribution characteristics in large animal models is essential. In conclusion, this study offers new insights into tumor immunotherapy and presents a promising strategy for future clinical applications.

## 4 Conclusion

In this study, we successfully developed a multifunctional biomimetic nanodelivery platform, PGFP+, offering an innovative strategy for tumor therapy by synergistically integrating targeted peptide‐mediated PD‐L1 protein degradation and photothermal immune regulation strategies. This platform employs platelet membrane‐functionalized nanocarriers to precisely target the tumor microenvironment, while thermosensitive liposomes enable controlled drug release under mild local thermal conditions. Fluorinated therapeutic peptides effectively inhibit the binding of TMUB1‐PD‐L1 and promote PD‐L1 degradation through the ubiquitin‐proteasome pathway. In addition, the photothermal effect mediated by the photosensitizer ICG can induce immunogenic cell death, thereby reshaping the immunosuppressive microenvironment. Collectively, this study presents a novel and promising peptide‐based immunotherapy approach aimed at enhancing cancer immunotherapy through the optimized delivery of anti‐tumor immune peptides alongside immune regulation strategies using photothermal effects.

## 5 Experimental Section

### Materials

DPPC, DSPE‐PEG2000, MSPC, and cholesterol were purchased from AVT (Shanghai) Pharmaceutical Tech Co., Ltd. (Shanghai, China). PTPR and FITC‐PTPR were purchased from GL Biochem Co. Ltd. (Shanghai, China). ICG, PEI1800, heptafluorosuccinic anhydride, and methanol were purchased from Aladdin (Shanghai, China). Hoechst 33 342, DID (1,1′‐ octadecyl‐3,3′,3′‐ tetramethylindole dicarbocyanine, 4‐chlorobenzenesulfonate), LysoTracker Green, and LysoTracker Red were purchased from Beyotime Biotechnology (Shanghai, China). Calcein AM/PI reagent, RIPA buffer, ECL detection reagent, and dialysis membranes with molecular weights of 1000 and 3500 Da were purchased from Soleibao (Beijing, China). The TRIzol reagent was purchased from Invitrogen (USA). The universal SYBR Green Supermix qPCR kit and iScript cDNA synthesis kit were purchased from Bio‐Rad (Hercules, CA, USA). The anti‐PDL1 antibodies were purchased from Abcam (Cat# ab213480; Cat# ab205921, Cambridge, UK). The human CD3/CD28 T cell activator was purchased from STEMCELL Technologies (Cat#10 970, Vancouver, Canada). Mouse CD3 (Cat# BE0001‐1‐1MG) and CD28 (Cat# BE0015‐1‐1MG) T cell activators were purchased from Bioxcell (West Lebanon, USA). Horseradish peroxidase (HRP)‐conjugated secondary antibody was purchased from Proteintech Group, Inc. (Chicago, IL, USA). The CCK‐8 and TUNEL detection kits were purchased from Abbkine (Wuhan, China). Antibodies against CD4‐PB (Cat# 100 531) and PD‐L1‐PE (Cat# 124 308) were purchased from BioLegend (San Diego, CA, USA). Antibodies against CD3‐FITC (Cat# 11‐0032‐82), CD44‐APC (Cat# 17‐0441‐82), CD62L‐PerCP‐Cyanine5.5 (Cat# 45‐0621‐80), CD11c‐FITC (Cat# 11‐0114‐82), CD80‐APC (Cat# 17‐0801‐81), CD86‐PE (Cat# 12‐0862‐81), CD25‐PE (Cat# 12‐0251‐81), Foxp3‐FITC (Cat# 11‐5773‐82), and CD8‐PE (Cat# 12‐0081‐82) were purchased from eBioscience (California, USA).

### Synthesis of PGFP

PGFP was synthesized using microfluidic mixing.^[^ ^66^ ^]^ Briefly, DPPC, cholesterol, MSPC, and DSPE‐PEG2000 were dissolved in ethanol at molar ratios of 70:22.5:2.5:5. PTPR (GFTATPPAPDSPQEP) or PTPRF (GFTATPPPDSPQEP/PEIF) were dissolved in an aqueous solution, with microfluidic mixing performed at a flow rate of 20 mL min^−1^ and a temperature of 45 ℃, using a microfluidic device (INano L, Micro&Nano Technology Co., Ltd., Shanghai, China). The ICG photosensitizers or DID (1,1′‐Dioctadecyl‐3,3,3′,3′‐Tetramethylindodicarbocyanine, 4‐Chlorobenzenesulfonate Salt) fluorescent dyes were incorporated during the lipid dissolution stage to form thermosensitive liposomes labeled with either photosensitizers or fluorescent dyes. Unencapsulated ICG and PTPR were removed via overnight dialysis at 4 ℃ (MWCO = 3500 Da). Platelet membranes—obtained by freezing and thawing in liquid nitrogen for ten cycles, followed by ultrasonic treatment—were added to the mixture. The resulting solution was sequentially passed through 400, 200, and 100 nm polycarbonate membranes to form platelet membrane‐modified liposome nanoparticles.^[^ ^60^ ^]^ Finally, the size distribution and polydispersity index (PDI) of the nanoparticles were evaluated via dynamic light scattering using a Zetasizer Nano ZSP (Zetasizer Ultra, UK). The morphology and size distribution of the nanoparticles were observed via transmission electron microscopy (TEM; JEM‐1400flash, JEOL).

### Synthesis of PEIF

PEIF was synthesized following a modified protocol based on previous literature.^[^ ^67^ ^]^ Briefly, PEI 1800 and heptafluorobutyric anhydride were dissolved in methanol at a molar ratio of 1:4. Triethylamine was then added to the mixture, which was subsequently stirred at room temperature for 48 h. The resulting mixture was dialyzed against double‐distilled water using a dialysis membrane with a molecular weight cutoff (MWCO) of 1000 Da. A white powder was obtained after vacuum freeze‐drying. The presence of fluorinated bonds was verified by ^19^F NMR analysis.

### Cellular Uptake of PGFP

The human breast cancer cell line MDA‐MB‐231 (CRM‐HTB‐26; RRID: CVCL_0062) and mouse breast cancer cell line 4T1 (CRL‐2539; RRID: CVCL_0125) were obtained from the National Infrastructure of Cell Line Resources (China). Before experimentation, all cell lines were screened for mycoplasma contamination and authenticated using short tandem repeat (STR) profiling.

The cellular uptake of PGFP was evaluated using confocal microscopy and flow cytometry. 4T1 cells were cultured in confocal dishes for 24 h, followed by treatment with PGFP for 1, 3, 6, and 8 h. Lysosomes and endosomes were labeled using the LysoTracker Red or LysoTracker Green probes. The nuclei were stained with Hoechst 33 342 for 10 min, after which the cells were washed and fixed with 4% paraformaldehyde for 15 min. The samples were examined using a confocal laser scanning microscope (Zeiss LSM 900, Germany) or a rotating disc confocal laser microscope (Olympus IXplore SpinSR, Japan). For flow cytometry analysis, cultured 4T1 cells were treated with PBS, PTPR‐FITC, or various concentrations of PGFP for 6 h. MDA‐MB‐231 cells were treated with different concentrations of LPG, GP‐FITC, GFP‐FITC, or PGFP‐FITC for 6 h. After treatment, the cells were washed with PBS and analyzed using a flow cytometer (BD FACS Canto II, BD Biosciences).

### Animals

All animal experiments were conducted according to the experimental protocol ZJU20240674, which was specifically approved by the Animal Care and Use Committee of Zhejiang University School of Medicine. Female BALB/c mice weighing 18–22 g and aged 6–8 weeks were procured from the Shanghai Experimental Animal Center and housed under pathogen‐free conditions (12‐h light/dark cycle, 22 ℃, 40% humidity). The mice had ad libitum access to standard rodent chow and water.

### Anti‐Tumor Study in 4T1 Tumor Model

Anti‐tumor studies were performed on a 4T1 tumor model to assess the combined therapeutic effects of photothermal therapy and the PTPR therapeutic peptide. At the time of tumor inoculation, 1 × 10^6^ 4T1 cells were injected subcutaneously into the mammary fat pads of female BALB/c mice. Once the primary tumor volume reached ≈50 mm^3^, the tumor‐bearing mice were randomly divided into five groups and treated with 250 µL of saline, vehicle, PTPR, PGF+, or PGFP+ via the tail vein. The symbol “+” indicates that the mice underwent 15 min of 808 nm near‐infrared laser irradiation, with the temperature maintained between 42–43 °C. The tumor volume and body weight were recorded every 2 d throughout the treatment period. The tumor volume was calculated using the following formula:

(1) $$0.5\;\times \left(\mathrm{widt}h^{2}\times \mathrm{length}\right)\left(\mathrm{unit}:mm^{3}\right)$$

### Inhibition of Distal Tumor Growth

The remote therapeutic effect of the PGFP+ strategy on distal tumors was assessed using a 4T1 tumor model. For primary tumor inoculation, 4T1 cells (1 × 10^6^) were resuspended in PBS and injected subcutaneously into the left mammary fat pads of female BALB/c mice. Simultaneously, 4T1 cells (5 × 10^5^) were resuspended in PBS and injected subcutaneously into the right mammary fat pad to establish distal tumors. Once the volume of the left tumor reached ≈50 mm^3^, the mice were randomly divided into five groups (n = 5) and treated as previously described. Throughout the treatment period, the volumes of both the distal and primary tumors were recorded.

### Anti‐Metastatic Effect

To evaluate the anti‐metastatic therapeutic effect of PGFP+, tumor growth inhibition studies were conducted using a 4T1 tumor model. 4T1 cells (5 × 10^5^) suspended in PBS were injected into the subcutaneous fat pad of the left mammary glands of mice. The tumor‐bearing mice were randomly divided into five groups and treated with saline, vehicle, PTPR, PGF+, or PGFP+ via tail vein injections on days 7, 10, 13, 16, and 19. The laser irradiation group received routine 42–43 ℃ laser treatment. On day 20, 4T1 cells (1 × 10^6^) were injected into the tail veins of the mice. The mice were euthanized on day 34, and their spleens, lungs, and primary subcutaneous tumors were isolated. The establishment of 4T1 lung tumors and their anti‐tumor therapeutic effects were assessed via H&E staining.

### Statistical Analysis

Comparisons between two groups were performed using a two‐tailed Student's t‐test, while comparisons among multiple groups were performed using one‐way or two‐way analysis of variance (ANOVA), followed by Tukey's post hoc test. Results were considered statistically significant at *P* < 0.05. Results are expressed as *P < 0.05, **P < 0.01, and ***P < 0.001. Data analysis and graphical representations were performed using Prism 9.4.0 or Origin 2021.

## Conflict of Interest

The authors declare no conflict of interest.

## Author Contributions

M.T., C.S., G.C., and X.S. contributed equally to this work. A.L., M.Z., X.L., and K.W. conceived and designed the research. M.T., C.S., G.C., and X.S. performed most of the biochemical, molecular experiments, with assistance from F.L., L.Z., G.C., X.C., M.Y., Y.C., Y.W., Y.C., S.Y., and W.W. M.T. and C.S. performed in vivo experiments. M.Z., X.L., K.W., J.S., and Q.Y. contributed to the discussion, data interpretation, and the provision of experimental technical guidance. A.L., M.Z., X.L., and K.W. edited the manuscript. A.L., M.T., and C.S. wrote the manuscript.

## Supporting information



Supporting Information

## Data Availability

The data that support the findings of this study are available from the corresponding author upon reasonable request.
