# Supplementary material for: PD‐L1‐Targeting Biomimetic Photoresponsive Thermosensitive Liposomes for Triple‐Negative Breast Cancer
Source: Adv Sci (Weinh). 2025 Aug 11;12(41):e06841. doi: 10.1002/advs.202506841 (PMC12591182; doi:10.1002/advs.202506841)
Supplement: Supplementary file 1 — Supporting Information [file ADVS-12-e06841-s001.pdf]

# PD-L1-Targeting Biomimetic Photoresponsive Thermosensitive Liposomes for Triple-Negative Breast Cancer

*Manman Tan[1#], Chengyu Shi[1,2#], Guangyi Chi[1#], Xinwan Su[1#], Fangzhou Liu[1], Linyu Zhu[1], Guangqian Cheng[1], Xiangyi Chen[1], Meng Yu[1], Yijian Chen[1], Ying Wang[1], Yu Chen[1], ShuLing Yan[1], Wenfei Wu[1], Qingfeng Yan[1], Jianzhong Shao[1], Kai Wang[3,4*], Xiangrui Liu[5,6,7*], Min Zhou[5,7,8*] and Aifu Lin[1,3,5,7,9,10*]*

[1] MOE Laboratory of Biosystem Homeostasis and Protection, College of Life Sciences, Zhejiang University, Hangzhou, Zhejiang, 310000, China.

[2] Key Laboratory of RNA Science and Engineering, Institute of Biophysics Chinese Academy of Sciences, Beijing, 100101, China.

[3] Department of Respiratory and Critical Care Medicine, Center for Oncology Medicine, the Fourth Affiliated Hospital of School of Medicine, and International School of Medicine, International Institutes of Medicine, Zhejiang University, Zhejiang, 322000, China.

[4] Zhejiang Key Laboratory of Precision Diagnosis and Treatment for Lung Cancer, Yiwu, 322000, China

[5] Key Laboratory of Cancer Prevention and Intervention, The Second Affiliated Hospital, Zhejiang University School of Medicine, Hangzhou, Zhejiang, 310000, China.

[6] Department of Pharmacology, and Department of Gastroenterology of the Second Affiliated Hospital, Zhejiang University School of Medicine, Hangzhou, Zhejiang, 310000, China.

[7] Future Health Laboratory, Innovation Center of Yangtze River Delta, Zhejiang University, Jiashan, Zhejiang, 314102, China.

[8] Zhejiang University - University of Edinburgh Institute, Zhejiang University School of Medicine, Haining, Zhejiang, 314400, China.

[9] Cancer Center, Zhejiang University, Hangzhou, Zhejiang, 310000, China.

[10] Zhejiang Key Laboratory of Cell and Molecular Intelligent Design and Development, Zhejiang, 310000, China.

[#]These authors have contributed equally.

[*]To whom correspondence should be addressed: linaifu@zju.edu.cn; zhoum@zju.edu.cn; xiangrui@zju.edu.cn；kaiw@zju.edu.cn.

## Experimental Methods

### PTPR loading in liposomes

The drug loading of the PGFP complex was evaluated via fluorescence spectrophotometry. FITC-PTPR solutions were prepared at varying concentrations (10, 20, 40, 60, 80, 100, 150, 200, 350, 400, 500 μM). The fluorescence intensity was measured at excitation and emission wavelengths of 496 and 518 nm, respectively, to generate a standard curve. The initial fluorescence intensity (total drug concentration) of the liposomal suspension and the fluorescence intensity of the liposomes after preparation (encapsulated drug concentration) were measured. The encapsulation efficiency was calculated using the following formula: Encapsulation efficiency (%) = (Encapsulated drug concentration / Total drug concentration) × 100%.

### Cell viability assay

The cell viability of PGFP was evaluated via CCK8 assay and Calcein-AM/PI staining. The cells were seeded at a density of 4000 cells/well in 96-well plates and incubated for 24 h. The cells were then treated with different concentrations of PBS, PEI 2500, PEI 1800, PEIF1800, or PGFP for 24 h. Subsequently, 10 μL of the CCK-8 solution was added to each well; after 4 h of incubation, the absorbance was measured at 450 nm using a microplate reader (SpectraMax M5, Molecular Instruments, USA). To evaluate the viability of laser-irradiated PGFP, the cells were exposed to either a laser power of 1.0 W/cm$^2$ or varying laser powers before the addition of the CCK8 solution. In the Calcein-AM/PI staining, after cell incubation and adhesion, live cells (green) were stained with Calcein-AM, while dead cells (red) were stained with PI at 37 ℃ for 15 min. After washing, the cells were imaged and documented using a laser confocal microscope (Zeiss 900, Germany).

### In vitro photothermal effects

To evaluate the photothermal performance of PGFP, ICG-loaded PGFP samples at varying concentrations were placed in 200 μL microtubes and irradiated with an 808-nm laser at a power density of 1.0 W/cm$^2$. The temperature changes were monitored using an infrared thermal imaging camera (FLIR E50, USA). To assess the photothermal response under mild irradiation conditions, PGFP containing 60 μM ICG was irradiated at power densities of 0.33 and 1.0 W/cm$^2$. PBS was used as a control, and the cells were irradiated at 1.0 W/cm$^2$. Photothermal stability was evaluated by subjecting PGFP (80 μM ICG) to five on/off irradiation cycles using an 808-nm laser at a power density of 1.0 W/cm$^2$. The temperature

fluctuations were continuously recorded using an infrared thermal imaging camera (FLIR E50, USA).

**Cell lysis and immunoblotting**

Cells were washed with PBS and lysed using RIPA buffer supplemented with protease inhibitors. The lysates were subjected to SDS-PAGE (12% or 10% Bis-Tris gel) and subsequently transferred onto a nitrocellulose membrane. The membrane was blocked with TBS-T containing 5% non-fat dry milk, followed by overnight incubation with the primary antibody at 4 °C. After triplicate washing with TBS-T, the membrane was incubated with the HRP-conjugated secondary antibody for 1 h at room temperature and developed using ECL detection reagents.

**RT-qPCR**

The TRIzol reagent was used for RNA extraction according to the manufacturer's instructions. Total RNA was reverse-transcribed into cDNA using an iScript cDNA synthesis kit. Quantitative analysis of the target genes was performed using the Universal SYBR Green Supermix qPCR kit. GAPDH was used as the reference gene for relative quantification normalization, with details of the primer sequences provided in Supplementary Table 1.

**T Cell killing assay**

After euthanizing the mice, the left abdomen was disinfected with 75% alcohol, and the spleen was removed and ground. The spleen cells were collected via centrifugation at 500g for 3 min at room temperature. The lysis buffer (1 × RBC) was added, followed by incubation at room temperature for 5 min. The lysis buffer was then neutralized by adding an equal volume of RPMI 1640 medium, followed by centrifugation at 500 × g for 3 min. The resulting T cells were resuspended in T cell culture medium, transferred to a 12-well plate pre-coated with CD3 and CD28 antibodies (3 μg/mL), and incubated at 37 ℃ for 48 h. Subsequently, the activated T cells were cultured in T cell culture medium containing IL-2 (10 ng/mL; PeproTech). The cancer cells were seeded and allowed to adhere overnight. Subsequently, the activated T cells were added at an effector-to-target (E:T) ratio of 3:1 and co-cultured for 48 h. After incubation, the cells were washed with PBS to remove T cells and debris, followed by crystal violet staining to quantify the live cancer cells.

For the MDA-MB-231 human tumor cell killing model, CD19-expressing MDA-MB-231 cells were pretreated with LPG, GP, GFP, or PGFP (containing 10 μM PTPR and 60 μM ICG) for 4 h, followed by

laser irradiation at 808 nm with a power density of 0.33 W/cm² for 5 min. After irradiation, the cells were co-cultured with anti-CD19 Jurkat T cells that had been previously activated using a Human CD3/CD28/CD2 T Cell Activator at an effector-to-target (E:T) ratio of 3:1 for 36 h. Cell viability was evaluated using crystal violet staining.

**Flow cytometry analysis of membrane PD-L1**

To evaluate membrane PD-L1 expression, cells were centrifuged at 1000 × g for 5 min, followed by incubation with the PD-L1-PE flow cytometry antibody diluted in PBS containing 0.5% BSA for 30 min at 4 ℃ in the dark. After PBS washing (×3), the cells were analyzed using a BD FACS Canto II flow cytometer, and the acquired data were processed using FlowJo 10.8.1 software. The PD-L1 membrane abundance was quantified based on the PD-L1-PE median fluorescence intensity (MFI) values.

**In vivo biodistribution of PGFP**

BALB/c mice with subcutaneous 4T1 tumors were intravenously injected with GFP-FITC, PGFP-FITC, or free FITC-PTPR. After 24 h, tissues from the heart, liver, spleen, lungs, kidneys, and tumors were collected, and ex vivo imaging was performed. Blood samples were collected every 5 min, 30 min, 1 h, 2 h, 4 h, 8 h, 12 h, 24 h, 48 h, and 72 h after injection for the pharmacokinetic studies of PGFP. Plasma was separated from the collected blood samples via centrifugation at 4000 rpm for 10 min, and the samples were analyzed using in vitro imaging.

**In vivo photothermal effects**

A total of 100 μL of 4T1 cells with a concentration of 1 × $10^7$ cells/mL was injected into the subcutaneous fat pad of the mammary gland of female BALB/c mice (age, 6 weeks; weight ~18–22 g). When the tumor volume reached ~50 mm³, 250 μL of phosphate-buffered PBS, blank liposomes (LIP), GFP, or PGFP was administered through the tail vein. Subsequently, the tumor site was irradiated with an 808-nm laser at an appropriate power density for 15 min. During laser irradiation, thermal images of the mice were recorded using an infrared thermal imager.

**Flow cytometry analysis of different immune cells**

To evaluate the immune response following the combined treatment, the spleens of mice from each group were surgically removed, and the splenic immune cells were isolated after performing red blood cell

lysis. The cells were then incubated with the anti-CD4-PB and anti-CD8-PE antibodies to quantify $CD4^+$ and $CD8^+$ T cells in the spleen via flow cytometry (BD FACSCanto II, USA). Regulatory T cells (Tregs) were analyzed by staining lymphocytes with anti-CD4-PB, anti-CD25-PE, and anti-Foxp3-FITC antibodies. Additionally, dendritic cells in the lymph nodes were analyzed by staining with the anti-CD11c-FITC, anti-CD80-PE, and anti-CD86-APC antibodies. All antibodies used in these experiments were diluted to approximately 1:200.

**Immunohistochemistry staining**

Paraffin-embedded sections were dewaxed in xylene, rehydrated in graded ethanol concentrations, and subjected to antigen retrieval by heating in citrate buffer. The tissue slices were incubated overnight at 4 ℃ with 3% BSA primary antibody. After rinsing with PBS, horseradish peroxidase-conjugated secondary antibodies diluted in 3% BSA were added, and the sections were incubated at room temperature for 1 h. The nuclei were stained with hematoxylin, blued with 0.5% aqueous ammonia, and sealed with neutral gum. Images were captured using a laser confocal microscope (LSM 900; Germany). After routine dewaxing of the paraffin sections, the cell nuclei were stained with hematoxylin, while the cytoplasm was stained with eosin. The sections were then sealed with neutral resin and images were collected and analyzed using a Zeiss LSM 900 microscope (Germany).

**Cytokine detection**

Serum isolated from treated mice was diluted and analyzed for TNF-α and IFN-γ using an ELISA kit, following manufacturer instructions.

**Immunofluorescence staining**

Tissue sections were immersed in citrate buffer and heated to 100 ℃ for 15 min to optimize antigen immunoreactivity. They were then permeabilized with 0.5% Triton X-100 for 10 min, blocked with 5% FBS at room temperature for 30 min, and subsequently incubated overnight at 4 ℃ with appropriately diluted primary antibodies. After rinsing with PBS, the sections were incubated with appropriately diluted secondary antibodies in the dark for 1 h. DAPI was applied to ensure complete coverage, and the cells were incubated in the dark at room temperature for 15 min. Finally, the slides were sealed with an anti-fading sealant, and fluorescent images were captured using an LSM 900 confocal microscope (Zeiss).

**TUNEL staining**

The dewaxed tissue sections were treated with 20 μg/mL DNase-free Proteinase K and incubated at 37 ℃ for 20 min. Subsequently, the TdT enzyme (2 μL) was added along with 48 μL of the fluorescent labeling solution and 50 μL of the TUNEL reaction mixture. The samples were incubated in the dark at 37 ℃ for 60 min, following which the nuclei were stained with DAPI at room temperature for 10 min, and images were acquired using a confocal laser scanning microscope.

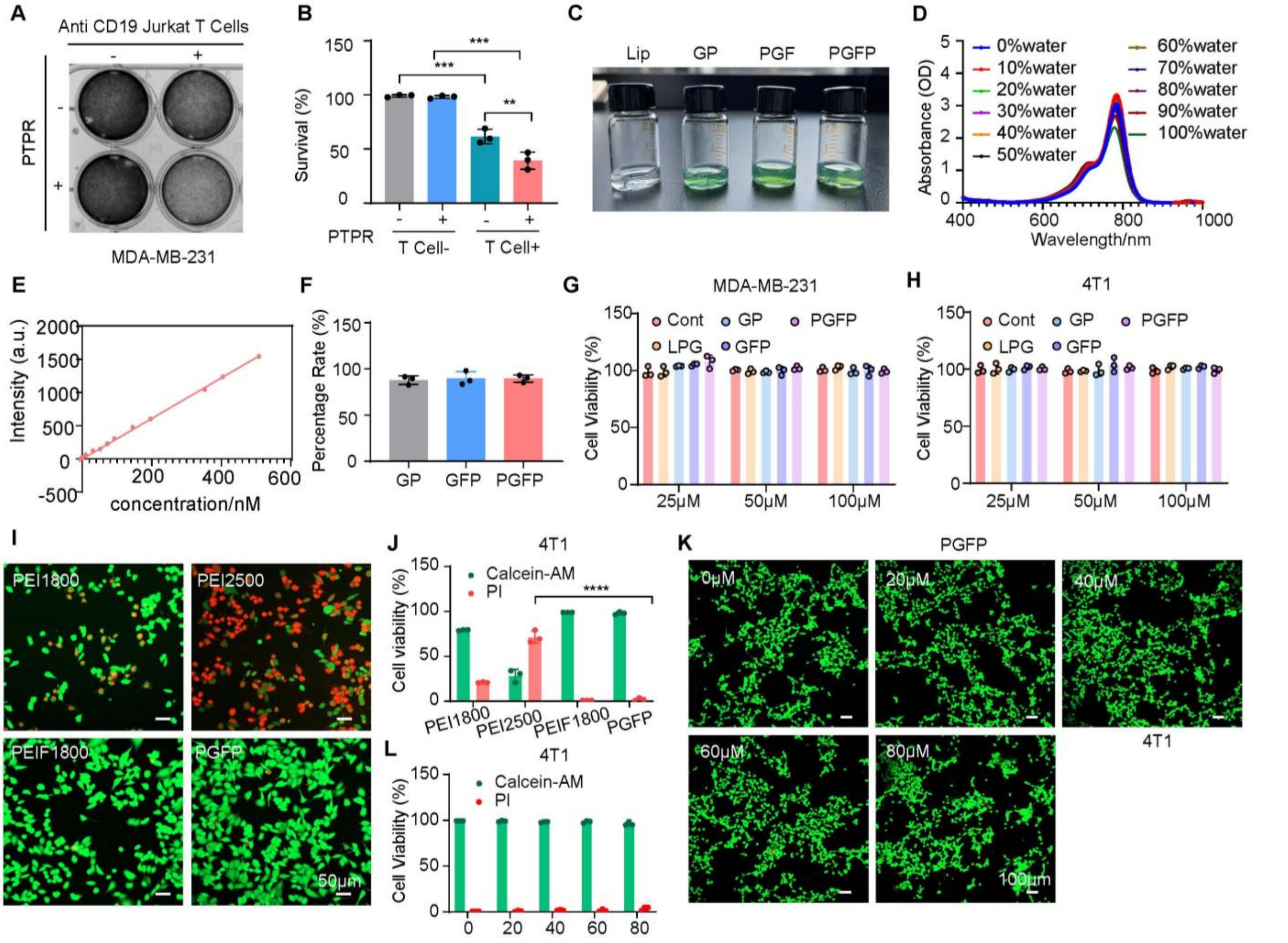


**Supplementary Figure 1. (A&B)** CD19-expressing MDA-MB-231 cells were treated with 20 µM PTPR for 12 h or left untreated, followed by co-culture with activated anti-CD19 Jurkat T cells at an effector-to-target (E:T) ratio of 1:3 for 48 h. Cell viability was assessed by crystal violet staining. Data are presented as mean ± SEM (n = 3), and statistical significance was determined using one-way ANOVA followed by Tukey's post hoc test. ***P<0.001, **P<0.01. **(C)** Solution images of Lip, GP, PGF, and PGFP. **(D)** UV-visible spectra of PGFP at different concentrations in pure water and ethanol. **(E)** Fluorescence measurements were performed using a spectrophotometer to detect FITC-PTPR solutions at various concentrations (10, 20, 40, 60, 80, 100, 150, 200, 350, 400, 500 µM). Fluorescence intensity was measured at an excitation wavelength of 496 nm and an emission wavelength of 518 nm, and a standard curve was plotted. **(F)** Fluorescence spectrophotometry was used to evaluate the drug loading capacity of GP, GFP, and PGFP on peptide drugs (n = 3). **(G&H)** CCK-8 assay of cell viability in MDA-MB-231 or 4T1 cells treated with varying concentrations of LPG, GP, GFP, or PGFP for 24 h. **(I&J)** Representative images of Calcein AM (green, live cells) and PI (red, dead cells) staining of 4T1 cells treated with 10 µg/mL PEI2500, PEI1800, PEIF1800, or PGFP (n = 3), along with quantitative analysis of cell viability and mortality using

ImageJ. Scale bar: 50 μm. **(K&L)** Calcein AM (green, live cells) and PI (red, dead cells) staining images and fluorescence statistical analysis of 4T1 cells treated with different concentrations of PGFP (n = 3). Scale bar: 100 μm.

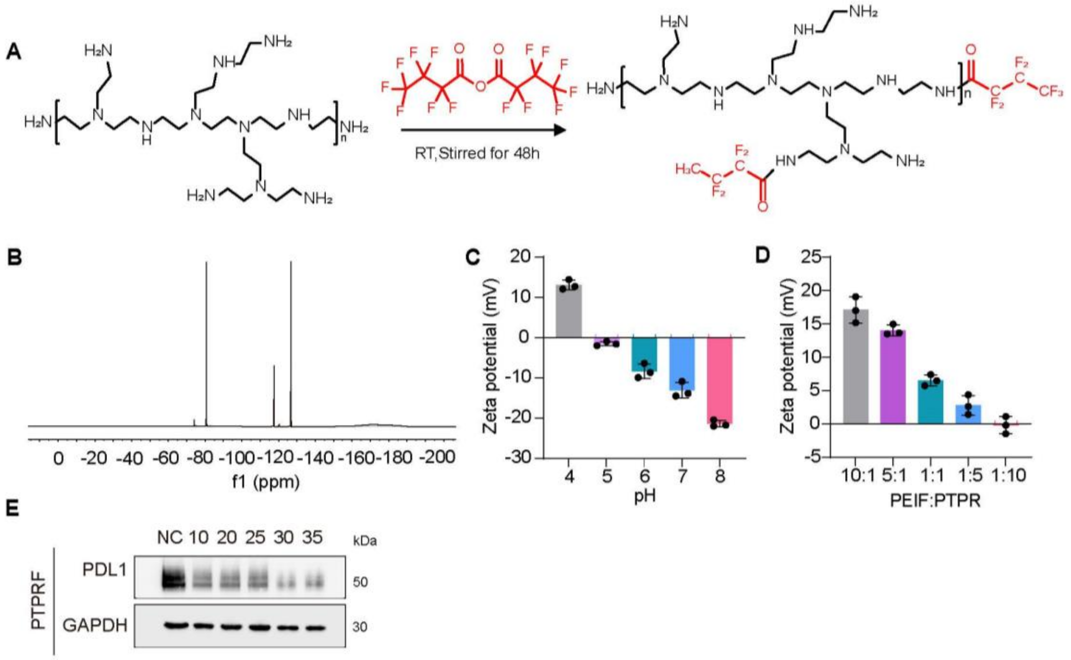


**Supplementary Figure 2. (A)** Schematic representation of the PEIF synthesis process. **(B)** Fluorine nuclear magnetic resonance (NMR) spectrum of PEIF. **(C)** Zeta potentials of PTPR at various pH levels. **(D)** Zeta potentials of different PEIF to PTPR ratios at pH 6.5. **(E)** Western blot analysis of PDL1 protein expression in 4T1 cells treated with varying concentrations of PTPRF.

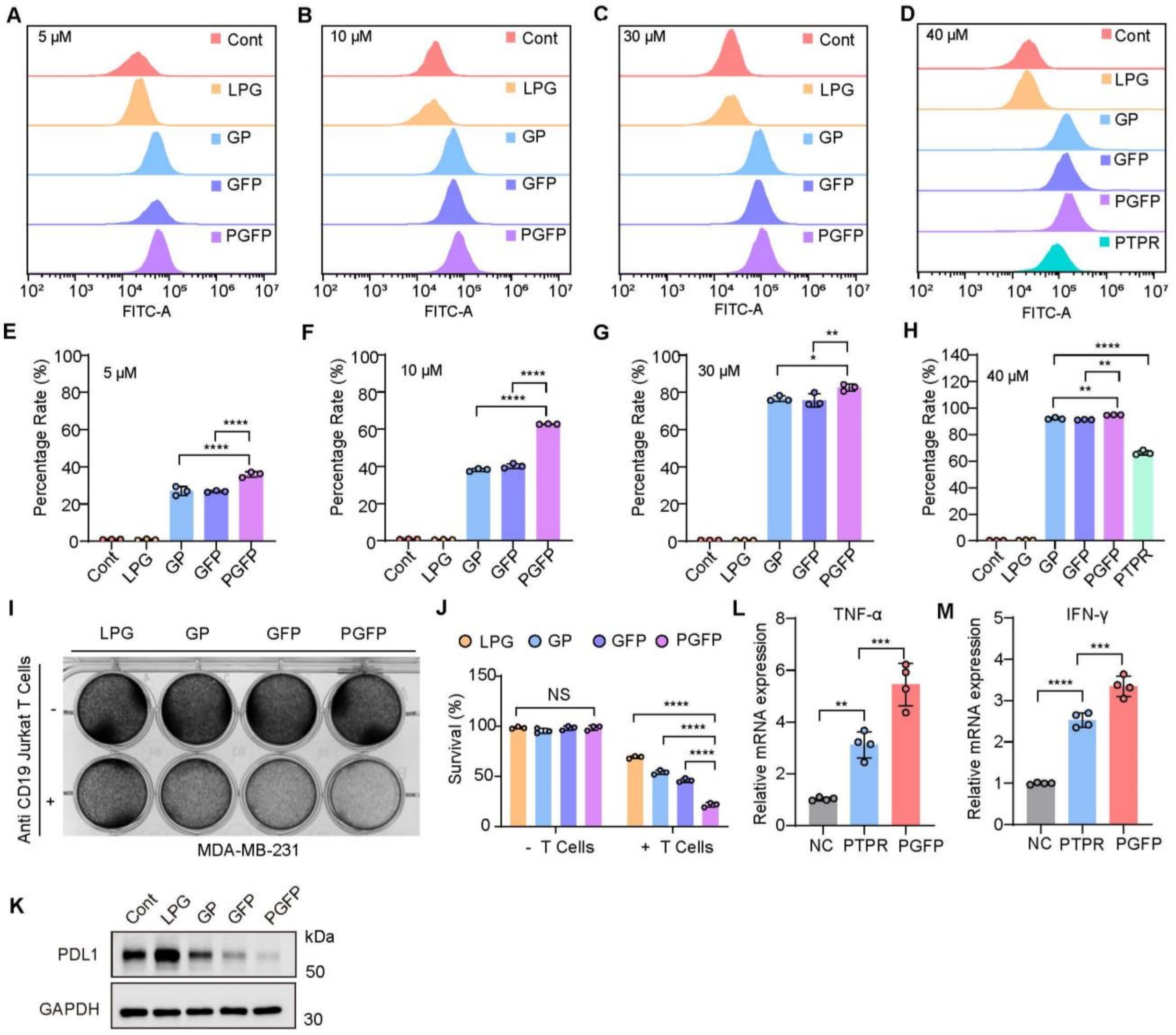


**Supplementary Figure 3. (A-D)** Flow cytometric analysis of the cellular uptake of LPG, GP, GFP, or PGFP at different concentrations in MDA-MB-231 cells. **(E-H)** Quantification of cellular uptake based on flow cytometry, expressed as the percentage of FITC-positive MDA-MB-231 cells treated with varying concentrations of LPG, GP, GFP, or PGFP. Statistical significance was determined using one-way ANOVA followed by Tukey's post hoc test. ****$P<0.0001$, ***$P<0.001$, **$P<0.01$, * $P < 0.05$. **(I&J)** CD19-expressing MDA-MB-231 cells were treated with 10 μM of LPG, GP, GFP, or PGFP for 12 h, followed by co-culture with activated anti-CD19 Jurkat T cells at an effector-to-target (E:T) ratio of 1:3 for 36 h. Cell viability was evaluated using crystal violet staining. Data are presented as mean ± SEM (n = 3), and statistical significance was analyzed using one-way ANOVA followed by Tukey's post hoc test. ****$P<0.0001$. **(K)** MDA-MB-231 cells were treated with LPG, GP, GFP, or PGFP for 4 h, followed by laser irradiation at 0.33 W/cm² for 5 min. After an additional 48 h of culture, PD-L1 protein expression was analyzed by Western blotting. **(L&M)** RT-qPCR analysis of TNF-α (K) and IFN-γ (L) expression in anti-

CD19 Jurkat T cells after PTPR or PGFP treatment of MDA-MB-231. Results are presented as mean ± SEM (n = 4), and statistical significance was assessed by one-way ANOVA followed by Tukey's post hoc test. ****P<0.0001, ***P<0.001, **P<0.01.

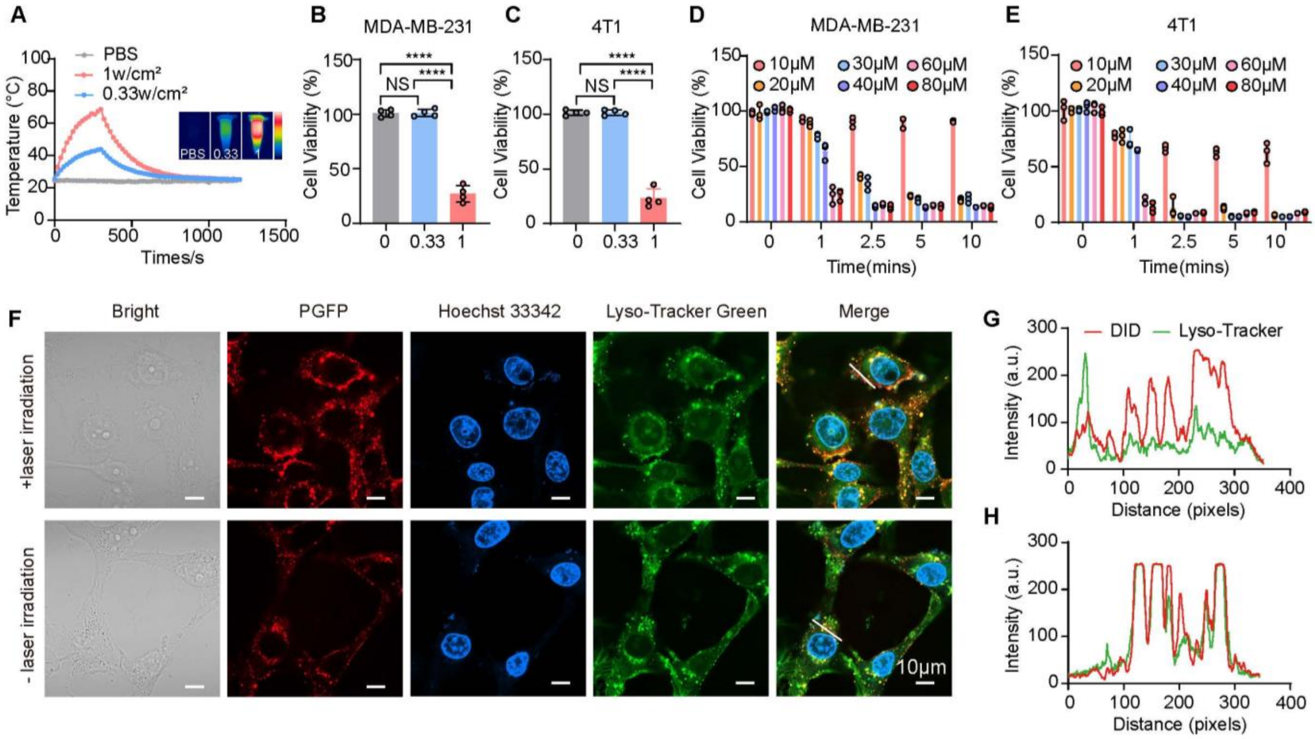


**Supplementary Figure 4. (A)** Temperature variation of PGFP during a single on–off laser irradiation cycle at different power densities**. (B&C)** Cell viability of MDA-MB-231 and 4T1 cells pretreated with PGFP (ICG 60 μM) followed by laser irradiation at 0.33 W/cm² or 1 W/cm². ANOVA with Tukey's test. ****P<0.0001. **(D&E)** Cell viability of MDA-MB-231 (C) and 4T1 (D) cells treated with PGFP at various ICG concentrations under 808 nm laser irradiation (1 W/cm²). **(F-H)** CLSM images of DID-PGFP complex incubated for 3 h with or without near-infrared irradiation and localization analysis curves of intracellular distribution. Scale bar: 10 μm.

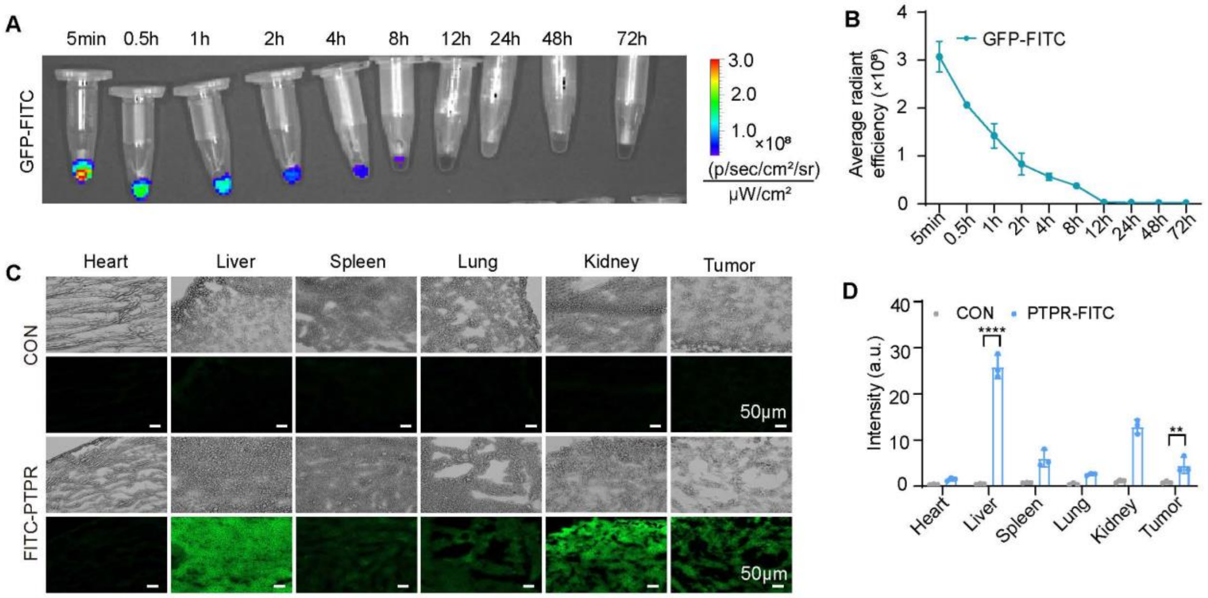


**Supplementary Figure 5. (A&B)** After intravenous injection of free GFP-FITC (n = 3) blood samples per time point), a time-dependent analysis of FITC fluorescence intensity in the blood was performed. **(C&D)** Representative fluorescence images and statistical analysis of fluorescence intensity in frozen sections of tumors and major organs were obtained 12 h after tail vein injection of PTPR-FITC (n = 3). Scale bar: 50 μm. Two-way ANOVA with Tukey's test. ****P<0.0001, **P<0.01.

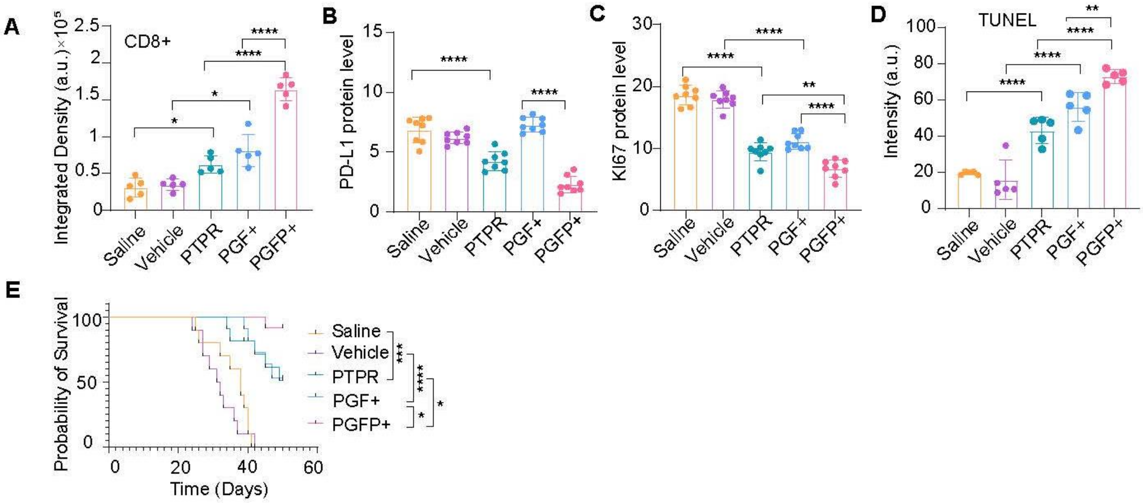


**Supplementary Figure 6. (A)** Statistical analysis of $CD8^+$ T cell immunofluorescence in tumor tissues from BALB/c mice following treatment (n=5). ANOVA with Tukey's test. ****P<0.0001, * P < 0.05. **(B)** Statistical analysis of PD-L1 protein expression in tumor tissues from BALB/c mice using immunohistochemistry after treatment (n=8). ANOVA with Tukey's test. ****P<0.0001. **(C)** Statistical analysis of Ki67 protein expression in tumor tissues from BALB/c mice using immunohistochemistry after treatment (n=8). ANOVA with Tukey's test. ****P<0.0001, **P<0.01. **(D)** Representative confocal fluorescence images and statistical analysis of TUNEL staining in tumor tissue sections from BALB/c mice following treatment. ANOVA with Tukey's test. ****P<0.0001, **P<0.01. **(E)** Survival analysis of mice bearing 4T1-derived tumors that were treated with saline, vehicle, PTPR, PGF+, or PGFP+. Each treatment group consisted of n=10 mice, and statistical significance was determined using the log-rank test. ****P<0.0001, ***P<0.001, * P < 0.05.

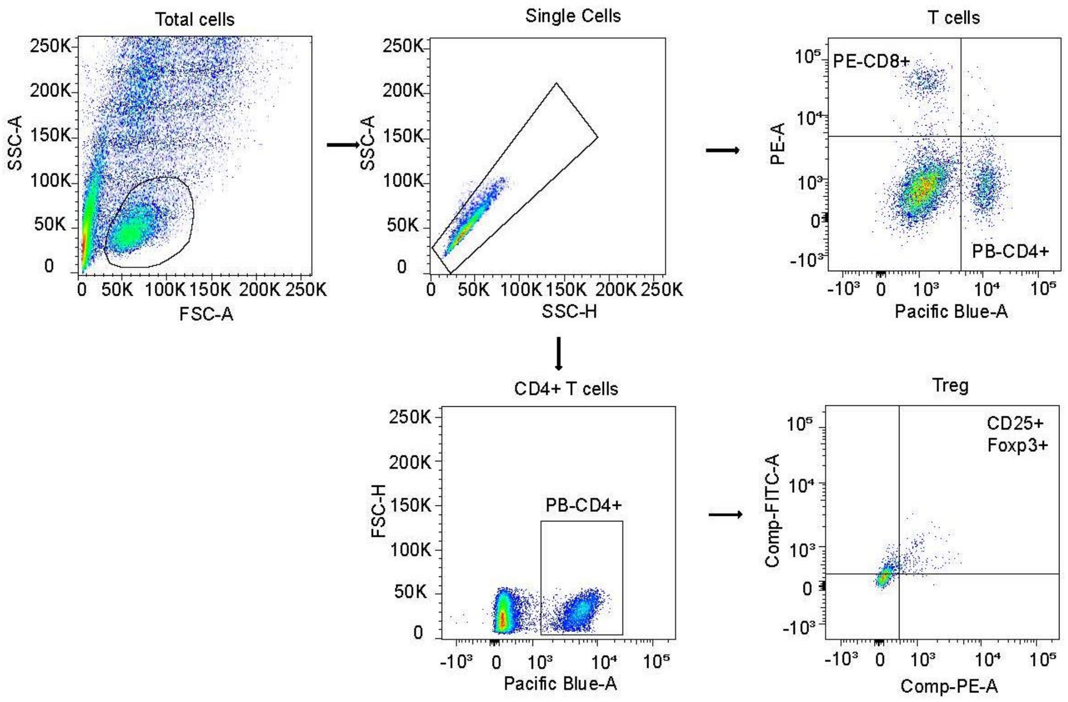


**Supplementary Figure 7.** The FACS gating strategy used to identify immune cell populations in the spleen was as follows: Lymphocytes were first gated based on forward scatter (FSC) and side scatter (SSC) parameters. The $CD8^+$ and $CD4^+$ T cell populations were distinguished by their surface expression of CD8 and CD4, respectively. Regulatory T cells (Tregs) were then identified within the $CD4^+$ T cell population by assessing the co-expression of Foxp3 and CD25. The quantitative results are presented in Figures 5K–N and 6I–M.

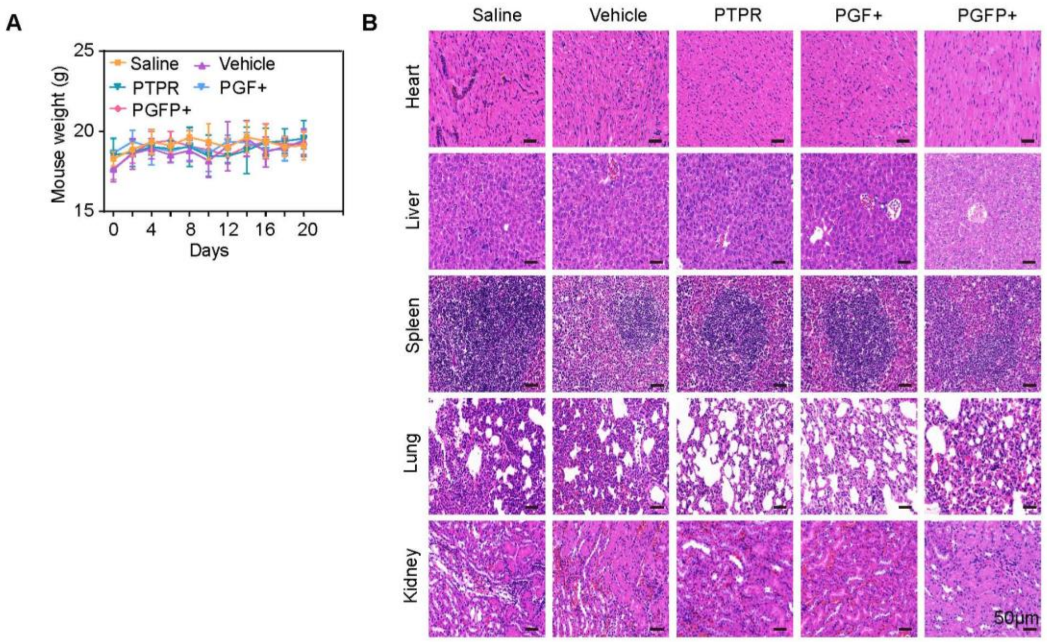


**Supplementary Figure 8. (A)** Body weight curves of mice during treatment (n = 5). **(B)** H&E staining of tissue sections from various organs of mice subjected to different treatments. Scale bar: 50 μm.

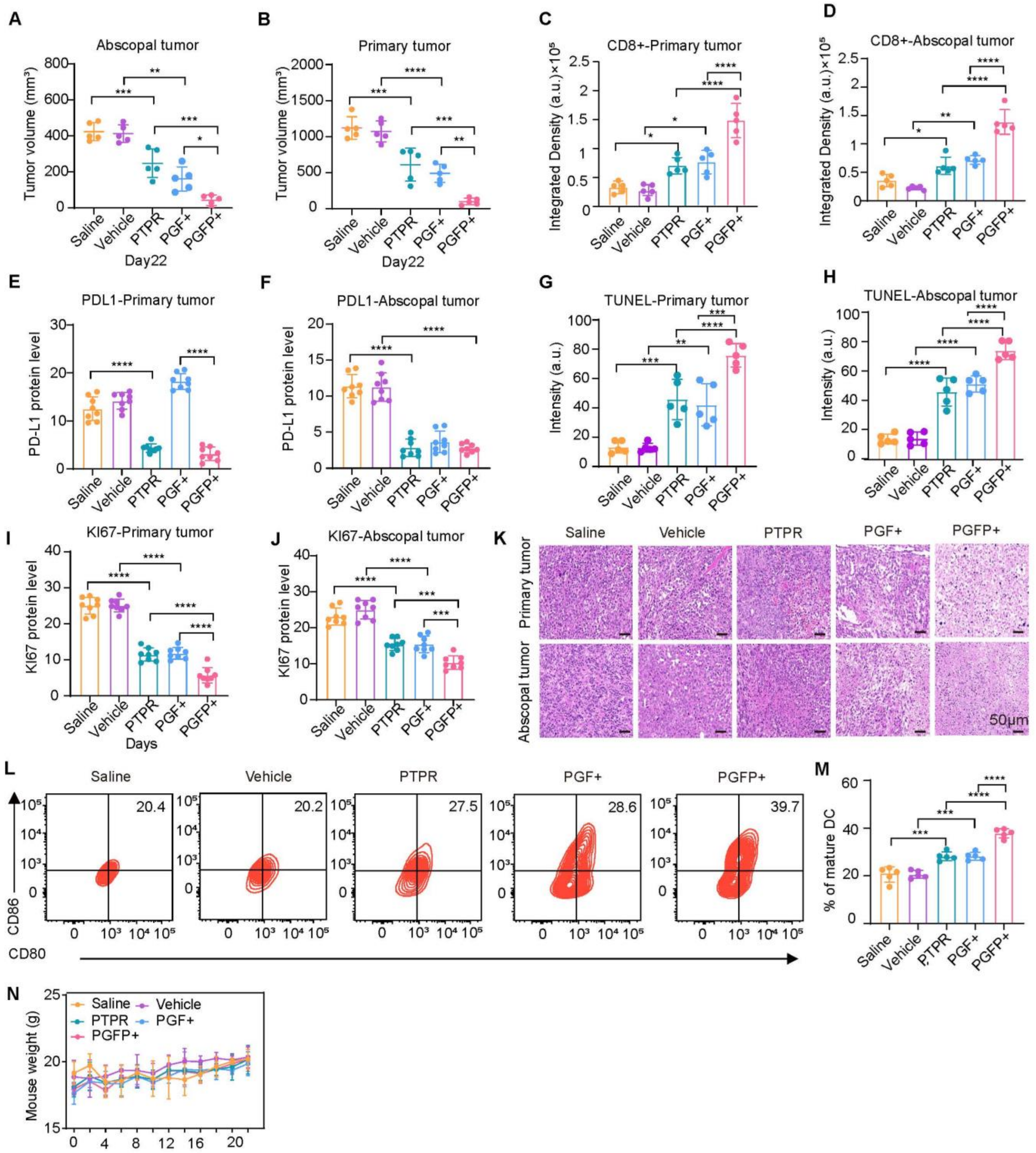


**Supplementary Figure 9. (A&B)** Statistical analysis of primary and distant tumor volumes in mice after treatment. Statistical significance was determined using one-way ANOVA followed by Tukey's post hoc test. ****P<0.0001, ***P<0.001, **P<0.01, * P < 0.05. **(C&D)** Representative images of $CD8^+$ effector T cells in primary and distant tumor tissue sections from mice after treatment (n=5). Integrated density (sum of fluorescence intensity) was quantified using ImageJ software, and statistical significance was evaluated using one-way ANOVA and Tukey's post hoc test. ****P<0.0001, **P<0.01, * P < 0.05. **(E&F)** Representative immunohistochemical images of PD-L1 protein expression in primary and distant tumor

tissue sections from mice after treatment (n=8). The percentage area (%Area) was quantified using ImageJ software, and statistical significance was evaluated using one-way ANOVA and Tukey's post hoc test. ****P<0.0001. **(G&H)** Representative images of TUNEL staining in primary and distant tumor tissue sections from mice after treatment (n=5). Mean fluorescence intensity (calculated as mean gray value, Mean= IntDen/AreaMean = IntDen/Area) was quantified using ImageJ software, and statistical significance was evaluated using one-way ANOVA and Tukey's post hoc test. ****P<0.0001, ***P<0.001, **P<0.01. **(I&J)** Statistical analysis of Ki67 protein expression from representative immunohistochemical images in primary and distant tumor tissue sections from mice after treatment (n=8). The percentage area (%Area) was quantified using ImageJ software, and statistical significance was evaluated using one-way ANOVA and Tukey's post hoc test. ****P<0.0001, ***P<0.001. **(K)** Representative images of primary and metastatic tumor tissue sections stained with H&E. Scale bar: 50 μm. **(L&M)** Flow cytometric analysis of DCs maturation in lymph nodes of mice following different treatments. Results are presented as the mean ± SEM (n = 5), with statistical significance assessed using one-way ANOVA followed by Tukey's post hoc test. **(N)** Body weight curves of mice during treatment (n = 5). ****P<0.0001, ***P<0.001.

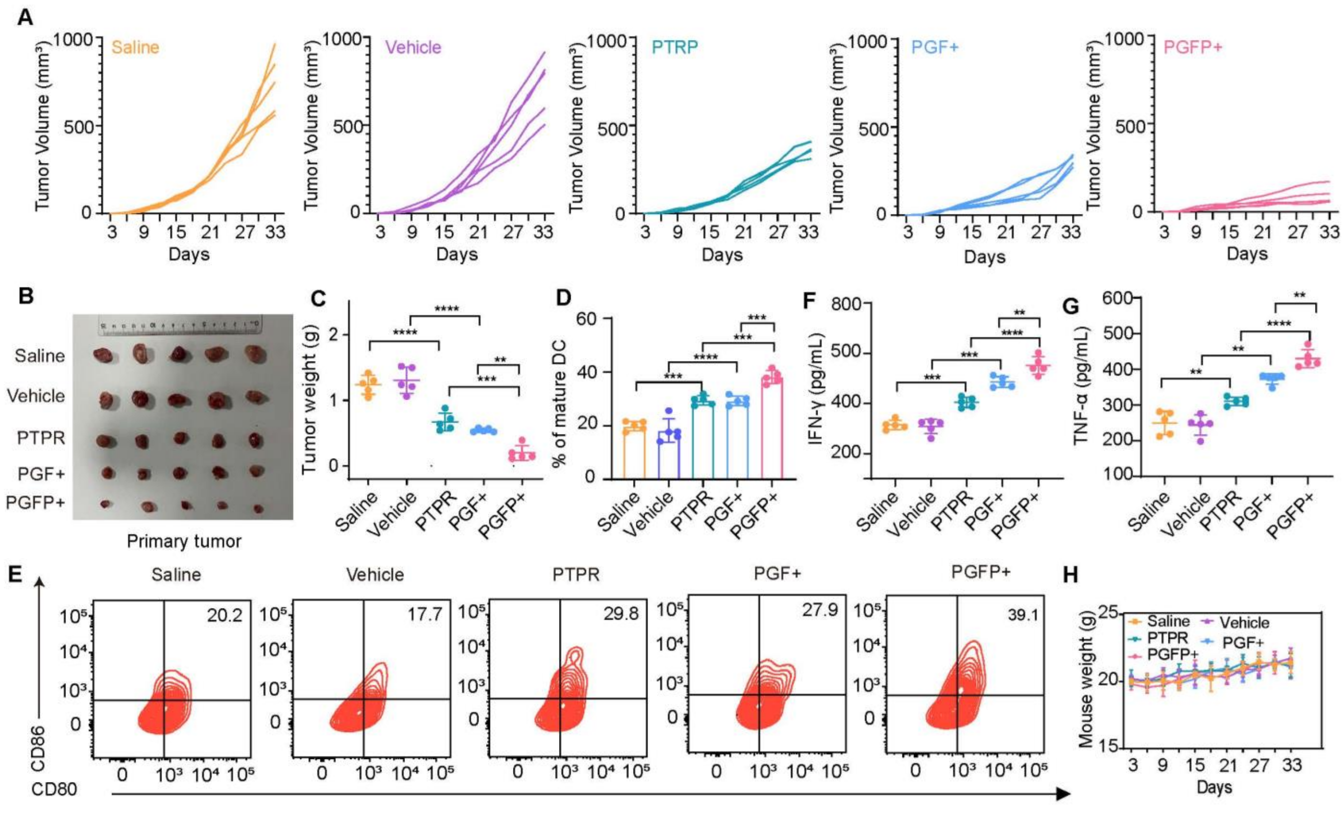


**Supplementary Figure 10. (A)** Primary tumor growth curves for individual mice in the BALB/c mouse model bearing 4T1 tumors after various treatments. **(B)** Representative images of subcutaneous fat pad tumors in the mammary glands of BALB/c mouse models (n = 5) bearing 4T1 tumors across different treatment groups. **(C)** Statistical analysis of in vitro tumor weights in mice following different treatments. ****P<0.0001, ***P<0.001, **P<0.01. **(D&E)** Flow cytometric analysis of DCs maturation in lymph nodes of mice following treatment. Data are expressed as the mean ± SEM (n = 5). Statistical significance was determined using one-way ANOVA followed by Tukey's post hoc test. ****P<0.0001, ***P<0.001. **(F&G)** Serum levels of IFN-γ and TNF-α in mice after the indicated treatments, presented as the mean ± SD (n = 5). Statistical significance was determined using one-way ANOVA followed by Tukey's post hoc test. ****P<0.0001, ***P<0.001, **P<0.01**.** **(H)** Body weight curves of mice during treatment (n = 5).

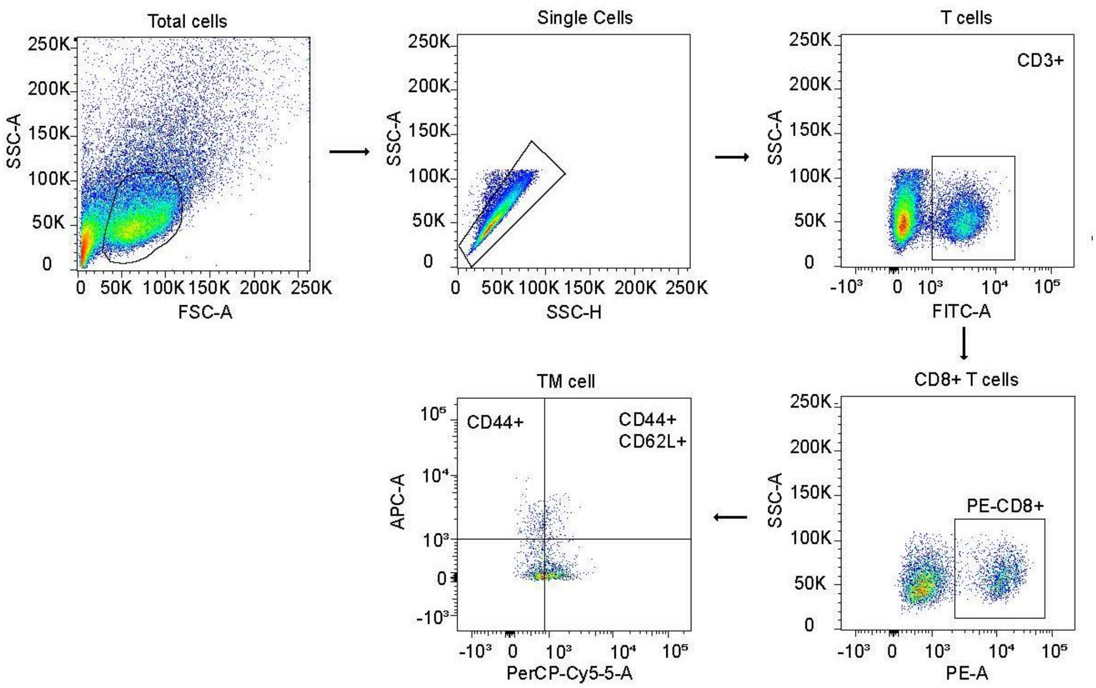


**Supplementary Figure 11.** Flow cytometric analysis was conducted to examine memory T cells in the spleen. Lymphocytes were initially gated based on forward scatter (FSC) and side scatter (SSC) parameters. T cell populations were then identified based on CD3 expression. Within the $CD3^+$ population, $CD8^+$ T cells were further gated using CD8 expression. Memory T cell subsets were distinguished by their differential expression of CD44 and CD62L. The results of the quantitative analysis are presented in Figures 7I–J.

| Oligonucleotides Resource | Sequence | Application |
|---|---|---|
| mGAPDH-F | 5'-TGATGACATCAAGAAGGTGGTGAA-3’ | RT-qPCR primer |
| mGAPDH-R | 5'-TGGGATGGAAATTGTGAGGGAGAT-3’ | RT-qPCR primer |
| mIFNγ-F | 5’-ACAGCAAGGCGAAAAAGGATG-3’ | RT-qPCR primer |
| mIFNγ-R | 5’-TGGTGGACCACTCGGATGA-3’ | RT-qPCR primer |
| mTNFα-F | 5’-CCCTCACACTCAGATCATCTTCT-3’ | RT-qPCR primer |
| mTNFα-R | 5’-GCTACGACGTGGGCTACAG-3’ | RT-qPCR primer |
| H-GAPDH- F | 5’-CCAAGGAGTAAGACCCCTGG-3’ | RT-qPCR primer |
| H-GAPDH- R | 5’-TGGTTGAGCACAGGGTACTT-3’ | RT-qPCR primer |
| H-IFNγ-F | 5’-TCGGTAACTGACTTGAATGTCCA-3’ | RT-qPCR primer |
| H-IFNγ-R | 5’-TCGCTTCCCTGTTTTAGCTGC-3’ | RT-qPCR primer |
| H-TNFα-F | 5’-CCTCTCTCTAATCAGCCCTCTG-3’ | RT-qPCR primer |
| H-TNFα-R | 5’-GAGGACCTGGGAGTAGATGAG-3’ | RT-qPCR primer |

**Supplementary Table 1.** Oligonucleotide sequences for qPCR.
